# Gut microbiota development, antibiotic resistome, and related perinatal factors in early infancy

**DOI:** 10.1128/msystems.00502-25

**Published:** 2025-07-31

**Authors:** Pianpian Fan, Jinqian Ma, Qianwen Shen, Youcheng Ouyang, Ting Zhang, Jian Shen, Zhong-Cheng Luo, Zhiwei Liu, Fengxiu Ouyang

**Affiliations:** 1 Ministry of Education and Shanghai Key Laboratory of Children's Environmental Health, Xinhua Hospital, Shanghai Jiao Tong University School of Medicine 91603 , Shanghai, China; 2 School of Exercise and Health, Shanghai University of Sport 659907, Shanghai, China; 3 Department of Neonatology, International Peace Maternity and Child Health Hospital, Shanghai Jiao Tong University School of Medicine 56694, Shanghai, China; 4 School of Food Science and Engineering, Jiangxi Agricultural University 682463, Nanchang, Jiangxi, China; 5 Department of Anesthesiology, East Hospital, Tongji University School of Medicine 481875, Shanghai, China; 6 Key Laboratory of Systems Biomedicine (Ministry of Education), Shanghai Center for Systems Biomedicine, Shanghai Jiao Tong University 12474, Shanghai, China; 7 Department of Obstetrics and Gynecology, Prosserman Centre for Population Health Research, Lunenfeld-Tanenbaum Research Institute, Mount Sinai Hospital, Institute of Health Policy, Management and Evaluation, University of Toronto 206712, Toronto, Ontario, Canada; Los Alamos National Laboratory, Los Alamos, New Mexico, USA

**Keywords:** gut microbiota, early life, infants, metagenomic sequencing, prospective study, assisted reproductive technology

## Abstract

Early life is a critical window for gut microbiota development and antibiotic resistome. We aimed to investigate the microbiota longitudinal change during the first 6 months of life and the differences associated with assisted reproductive technology (ART) treatment, mode of delivery, and infant sex. Gut microbiota was measured by metagenomic sequencing of fecal samples repeatedly collected within 3 days after birth, at 42 days, 3 months, and 6 months in 155 Chinese infants. Among infants born by vaginal delivery, the ACE, Chao, and Sobs indexes increased with age. Accordingly, the relative abundance of *Actinobacteria* phylum increased from 43% ± 37% (mean ± SD) to 57% ± 36% and *Proteobacteria* phylum decreased from 48% ± 36% to 17% ± 25%; and *Bifidobacterium* and *Klebsiella* genus increased and *Escherichia* genus decreased, from the first 3 days to 6 months. The dominant antibiotic resistance genes were *macB* and *msbA*. Boys and girls had similar features. Infants born by cesarean section exhibited a similar gut microbiota developmental trajectory, but with a slower increase in alpha diversity over time, and lower *Bacteroidetes* phylum and *Bacteroides* genus at each age point. Compared with non-ART infants, ART infants had slightly higher alpha diversity indexes of ACE, Chao, Sobs, Shannon, and Pielou’s evenness at age 42 days among infants born by vaginal delivery. Our findings confirm increasing diversity and composition evolution of gut microbiota in the first 6 months of life. Both modes of delivery and ART conception are associated with early gut microbiota alteration.

**IMPORTANCE:**

Gut microbiota plays an important role in various aspects of human health. Early life is a critical period for the development of gut microbiota. In this prospective study, we observed that the diversity and antibiotic resistance genes of gut microbiota increased gradually with age in the first 6 months of life. Boys and girls had similar features of gut microbiota. Cesarean section was associated with a lower abundance of *Bacteroidetes* phylum and *Bacteroides* genus. Compared with non-ART infants (spontaneous conception), ART infants had slightly higher alpha diversity indexes of ACE, Chao, Sobs, Shannon, and Pielou’s evenness at age 42 days among infants born by vaginal delivery. This study presents gut microbiota development with age, antibiotic resistome, and related perinatal factors in early infancy.

## INTRODUCTION

Early life is a critical window for gut microbiota development and antibiotic resistome change. Any aberrancies in this process may affect gut microbiota, which in turn post long-term health effects during childhood and beyond (1, 2), for example, increased risk of obesity (3), diabetes (4, 5), asthma (6), and allergic diseases (7, 8). Also, antimicrobial resistance has become an important global health challenge in the 21st century (9). The antibiotic resistance genes (ARGs) and resistome are core characteristics of the gut microbiome. The development of the infant’s resistome is linked to the evolution of the microbial composition (10). The spreading of microbial ARGs and the emergence it brought have become a critical global health issue (11). A better understanding is needed on gut microbiota development in early life to provide insight into how it develops and potential influential factors.

To the best of our knowledge, there have been limited data on longitudinal changes of the gut microbiota during early infancy, particularly scarce during the first 6 months of life—a rapid postnatal development phase for infant gut microbiota. A cross-sectional study reported that the phylogenetic diversity of the gut microbiota increased over age, along with an increase in the relative abundance of *Bacteroidetes* and a reduction in *Proteobacteria* species (12). Another study showed that mode of delivery, diet, and cessation of breastfeeding were associated with variation in diversity and composition of gut microbiota among 440 children at 5, 13, 21, and 31 weeks postpartum (13). More longitudinal studies are needed to examine the dynamic change of gut microbiota during this early life stage in newborns.

The initial colonization of infant gut microbiota may be vulnerable to perinatal factors (14). Of note, with assisted reproductive technology (ART) becoming more and more common practice in reproductive medicine clinics for infertile couples worldwide, newborns conceived through ART have been reaching more than 5 million in the world (15). While ART may have a potential impact on the maternal microenvironment and embryos, it could cause alternations of gut microbiota in the offspring. Few studies had explored the gut microbiota of ART infants (15, 16), focusing on the first 4 days after birth. The ART impact on gut microbiota remains unclear, especially in the first 6 months of life. Other factors, like mode of delivery (cesarean section and vaginal delivery), may also affect initial gut microbiota (14).

In this longitudinal study, we sought to characterize the evolution of gut microbiota in the first 6 months of life in boy and girl newborns, and its related perinatal factors including mode of delivery, ART, and infant sex at birth, 42 days, 3 months, and 6 months of age.

## MATERIALS AND METHODS

### Study participants and fecal sample collection

The study participants were part of the China National Birth Cohort study described previously (17). Briefly, pregnant women were recruited in the International Peace Maternity and Child Health Hospital (IPMCH), affiliated with Shanghai Jiao Tong University School of Medicine, between October 2017 and March 2019. These study participants were residents in Shanghai and received antenatal care, delivery, and postnatal follow-ups at the IPMCH.

Data on maternal and infant characteristics were abstracted from medical records, including maternal age, parity, weight before pregnancy, height, ART treatments (yes, no), gestational complications (gestational diabetes mellitus [GDM], gestational hypertension, and preeclampsia), mode of delivery, maternal antibiotics use during pregnancy, and intrapartum antibiotic prophylaxis.

Fecal samples were collected from a total of 198 full-term infants. We excluded infants of mothers with GDM (*n* = 31), gestational hypertension or preeclampsia (*n* = 8), infants who had used antibiotics within 3 days after birth (*n* = 1), and infants whose samples were collected within 2 weeks after antibiotic use (*n* =4). After these exclusions, a total of 155 mother-infant pairs were included in this study. There was no significant difference in probiotics use and breastfeeding rates between ART and non-ART newborns born by vaginal delivery or cesarean section. Three out of 22 (13.6%) infants in the ART group and 56 (38.4%) in the non-ART group had exclusive breastfeeding in the first 6 months.

### Fecal samples collection

To characterize the early gut microbiota in infants, fecal samples were collected from infants at the first 3 days, 42 days, 3 months, and 6 months during the period of 30 July 2018 to 31 January 2019. The fresh fecal sample was taken from the central part of the stool (i.e., close to the diaper side, instead of the surface), where it was least likely to be contaminated with urine, using a sterile spoon by research staff wearing sterile gloves, and then placed into a sterile tube. The samples were then immediately stored at −80°C until analysis. As for the diaper, if the infant had defecation during study follow-up visits at clinics, parents collected and handed it to research staff immediately within 5 minutes; if not, we gave sterile collection box and sterile gloves to the parents, who then collected the feces at home and delivered/shipped it to our research staff in low temperature (on ice) within half an hour. Our project paid the mailing cost.

The corresponding numbers of fecal samples successfully sequenced were 9, 136, 108, and 105, respectively, at the four time points of 3 days, 42 days, 3 months, and 6 months. There were 5 (3.2%) infants having fecal samples sequenced at all the four time points, 74 (46.8%) infants had fecal samples at 3 time points, 40 (25.8%) at 2 time points, and 36 (23.2%) infants at a single age point only. We compared the demographic and clinical characteristics between infants with and without fecal samples collected at each of the four age points (3 days, 42 days, 3 months, and 6 months); they were similar between the two groups at each age point. Moreover, we grouped the participants based on delivery method, gender, and ART factors. After grouping, the two groups still maintain homogeneity.

### DNA extraction and metagenomic sequencing of gut microbiota

Total genomic DNA was extracted from fecal samples using the QIAamp PowerFecal Pro DNA Kit (Qiagen, 51804). The metagenomic sequencing was performed on an Illumina Hiseq Xten (Illumina Inc., San Diego, CA, USA) with quality control (Text S1).

Representative sequences of the non-redundant gene catalog were annotated based on the NCBI NR database using blastp as implemented in DIAMOND v0.9.19 with an e-value cutoff of 1e^−5^ using Diamond (https://github.com/bbuchfink/diamond/, v0.9.19) for taxonomic annotations. The KEGG annotation was conducted using Diamond (https://github.com/bbuchfink/diamond/, v0.9.19) against the Kyoto Encyclopedia of Genes and Genomes database (http://www.kegg.jp/, version 94.2) with an e-value cutoff of 1e^−5^. Annotation of ARGs was conducted using Diamond (https://github.com/bbuchfink/diamond/, v0.9.19) against the CARD database (https://card.mcmaster.ca/home, version 3.0.9) with an e-value cutoff of 1e^−5^. The linear discriminant analysis (LDA) threshold for LDA effect size (LEfSe) analysis was set at 4.0, and the *P* value was set to <0.01 in this study.

We assessed alpha diversity by using six indices: ACE (abundance-based coverage estimator; richness estimator emphasizing low-abundance taxa), Chao1 (richness estimator), observed features (Sobs; absolute operational taxonomic unit [OTU] count), Shannon (combining richness and evenness), Simpson (dominance-weighted diversity), and Pielou’s evenness (J′ = H′/lnS; pure evenness measure). Alpha diversity comparisons were performed between infant sex, age, and mode of delivery with Wilcoxon tests. To visualize community differences, beta diversity was evaluated by principal coordinate analysis (PCoA) based on Bray-Curtis dissimilarity.

### Statistical analysis

Descriptive statistics were presented as mean ± standard deviation (SD) or *n* (%) for maternal and infant characteristics. Considering the effect of delivery mode on gut microbiota, we separately analyzed associations between early-life factors (infant age, sex, ART conception) with taxonomic composition, functional composition at KEGG level 3, and antibiotic resistome of gut microbiota in vaginally delivered infants and cesarean-delivered infants using linear regression models, with specific adjustments for each analysis (age models adjusted for sex and feeding pattern; sex models adjusted for age and feeding pattern; ART models adjusted for age, sex, and feeding pattern). Two-sided *P* values <0.05 were considered statistically significant. Data analyses were conducted in Statistical Analysis System (SAS), version 9.4 (SAS Institute, Inc., Cary, NC, USA).

## RESULTS

### Maternal and infants’ general characteristics

This study included 155 newborns and their follow-up data collected within 3 days after birth, at 42 days, 3 months, and 6 months. The mean maternal age was 31.6 ± 4.0 years at childbirth, and pre-pregnant body mass index (BMI) was 21.1 ± 3.2 kg/m^2^ (Table 1). Among 155 infants, 21 (13.6%) were conceived by ART treatments, among whom 14 (9.1%) were through *in vitro* fertilization and 7 (4.5%) through intracytoplasmic sperm injection. All were via frozen-embryo transfer. There were 40 (25.8%) delivered by cesarean section. All infants were born with a normal 5 minute Apgar score (≥8).

**TABLE 1:** Demographic and clinical characteristics in study infants (*n* = 155)

Characteristic	Mean ± SD or *n* (%)
Maternal age (years)	31.6 ± 4.0
Maternal BMI (kg/m^2^)	21.1 ± 3.2
<18.5	22 (14.3)
18.5–24	109 (70.8)
≥24	23 (14.9)
Parity, >1	49 (31.6)
Anemia	26 (16.8)
Group B streptococcus (+)	6 (3.9)
ART treatments (yes, %)	21 (13.6)
Cesarean section	40 (25.8)
Pre-mature rupture of membrane	25 (16.1)
Infant gender (male, %)	82 (52.9)
Gestational age at birth (weeks)	39.3 ± 0.9
Weight	
At birth (g)	3394.8 ± 372.6
At 42 days (kg)	5.3 ± 0.6
At 6 months (kg)	8.5 ± 1.0
Feeding pattern, birth to age 42 days	
Exclusive breastfeeding	72 (46.8)
Mixed feeding	69 (44.8)
Formula feeding	13 (8.4)
Feeding pattern, birth to age 3 months	
Exclusive breastfeeding	59 (41.6)
Mixed feeding	71 (50.0)
Formula feeding	12 (8.5)
Feeding pattern, birth to age 6 months	
Exclusive breastfeeding	55 (36.4)
Mixed feeding	96 (63.6)
Introduction of solid food before 6 months of age	
No	38 (26.0)
Yes	108 (74.0)

### Dynamics of gut microbiota with age and infant sex in newborns born by vaginal delivery

**Fig 1:**
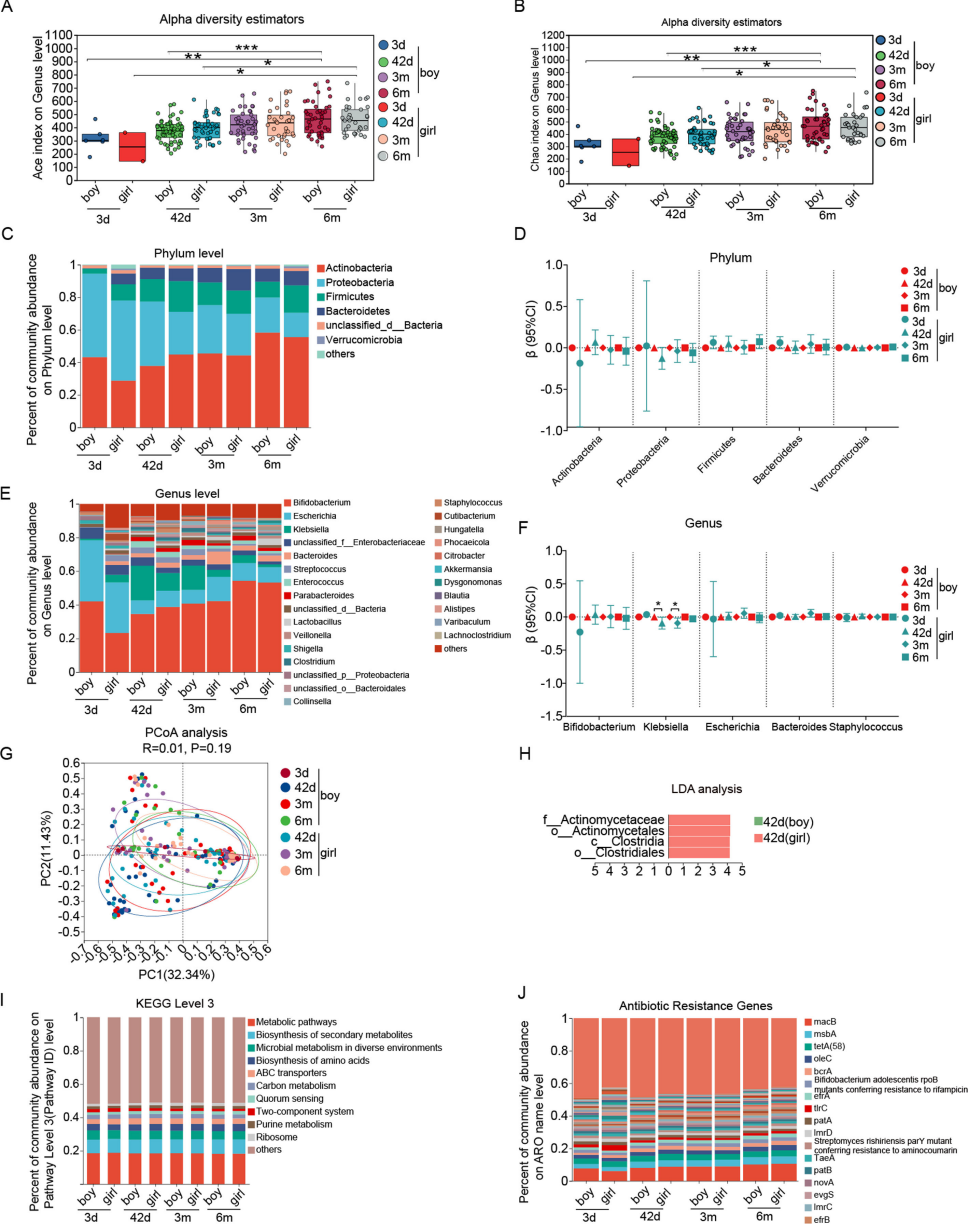
The alpha diversity on ACE index (**A**), Chao index (**B**), composition of phylum (**C and D**) and genus (**E and F**), β diversity (**G**), LDA (**H**), functional composition at KEGG level 3 (**I**), and antibiotics resistance genes (**J**) of gut microbiota from birth to 6 months of age in boy and girl infants born by vaginal delivery. Note: 3d, age 3 days; 42d, age 42 days; 3m, age 3 months; 6m, age 6 months. *P < 0.05, ** *P* < 0.01, *** *P* < 0.001.

With consideration of the impact of mode of delivery on newborn gut microbiota, we first analyzed the diversity and structure of gut microbiota with infant age and sex in vaginal and c-section delivery separately. Among infants delivered vaginally, as for alpha diversity, we observed indexes of ACE, Chao, and Sobs increased with age in both boys and girls (Fig. 1A and B; Fig. S1D), while the Simpson, Pielou’s evenness, and Shannon index (Fig. S1A through C) changed mildly with age, indicating an increase in richness but not in evenness. The top five dominant phyla were *Actinobacteria*, *Proteobacteria*, *Firmicutes*, *Bacteroidetes*, and *Verrucomicrobia*, and the top five dominant genera were *Bifidobacterium*, *Escherichia*, *Klebsiella*, *Bacteroides*, and *Streptococcus* during the first 6 months of life in both boys and girls (Fig. 1C through F; Tables S1 and S2). With increasing age from the first 3 days after birth to 6 months, the relative abundance of the *Actinobacteria* phylum increased from 43% ± 37% to 57% ± 36% (*P* trend = 0.01), *Bifidobacterium* from 40% ± 37% to 54% ± 36% (*P* trend = 0.005), and *Klebsiella* genus from 2% ± 3% to 3% ± 9% (*P* trend = 0.0005) increases, while the relative abundance of the *Proteobacteria* phylum decreased from 48% ± 36% to 17% ± 25% (*P* trend = 0.0006) and *Staphylococcus* genus from 1% ± 3% to 0.1% ± 0.3%, (*P* trend = 0.017) in infants (Tables S1 and S2). Boys and girls had similar features (Fig. 1C through F). Moreover, compared with infants at age 3 days, the relative abundance of *Escherichia* genus was 25% lower at age 42 days (95% confidence interval [CI]: 13%, 37%), 23% lower at 3 months (95% CI: 11%, 35%), and 24% lower at 6 months (95% CI: 12%, 36%) (Table S2).

The functional composition of the gut microbiota at KEGG level 3 was predominantly characterized by metabolic pathways, which accounted for 18%–19% across all ages in both boys and girls. These pathways exhibited mild changes over time (Fig. 1I; Tables S3), with an increasing trend observed in the relative abundance of biosynthesis of secondary metabolites (*P* trend = 0.009) and biosynthesis of amino acids (*P* trend = 0.003). Conversely, there was a decreasing trend in microbial metabolism in diverse environments (*P* trend = 0.001) and carbon metabolism (*P* trend = 0.035) (Fig. 1I; Tables S3). Among the top five dominant ARGs, the relative abundance of four ARGs increased over time in both boys and girls. Overall, it increased for *macB* (from 7.9% ± 4.7% to 10.4% ± 4.1%), *msbA* (from 2.6% ± 1.1% to 4.4% ± 2.2%), *oleC* (from 2.3% ± 0.9% to 3.0% ± 1.3%)*,* and *bcrA* (from 1.4% ± 0.8% to 2.3% ± 1.2%) and almost all the *P* trend <0.01 for test of trend with age (Fig. 1J; Table S4).

There were no significant differences in alpha diversity (ACE, Chao1, Sobs, Shannon, Simpson, and Pielou’s evenness) between boys and girls (Fig. 1A and B; Fig. S1). The PCoA showed that boys and girls had similar clusters in their gut microbiota (Fig. 1G). There were no significant differences in composition at phylum (Fig. 1C and D) and genus level (Fig. 1E and F), functional composition assessed at KEGG level 3 (Fig. 1I), and ARGs (Fig. 1J). LDA analysis identified several sex-differential taxa in infants only at 42 days of age (Fig. 1H), with a minor lower relative abundance of *Klebsiella* genus in girls at 42 days and 3 months of age (Fig. 1F).

### Dynamics of gut microbiota with age and infant sex in newborns born by C-section

**Fig 2:**
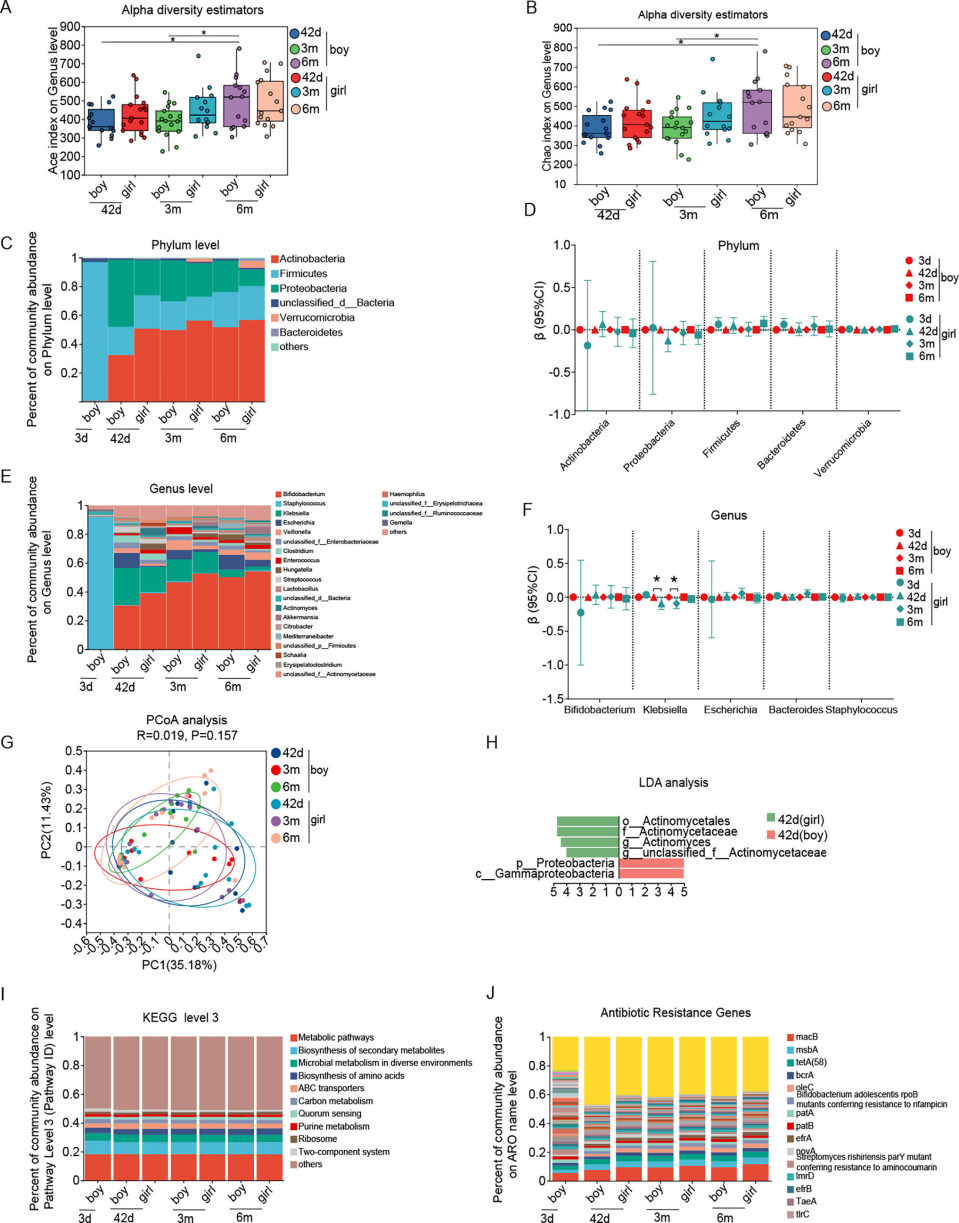
The alpha diversity on ACE index (**A**), Chao index (**B**), composition of phylum (**C and D**) and genus (**E and F**), β diversity (**G**), LDA (**H**), functional composition at KEGG level 3 (**I**), and antibiotics resistance genes (**J**) of gut microbiota from birth to 6 months of age in boy and girl infants born by cesarean section. Note: 3d, age 3 days; 42d, age 42 days; 3m, age 3 months; 6m, age 6 months. *P < 0.05.

We observed indexes of ACE, Chao, and Sobs increased with age only in boys (Fig. 2A and B; Fig. S2D), while there was no significant difference in the Simpson, Pielou’s evenness, and Shannon index (Fig. S2A through C), indicating an increase in richness but not in evenness in boys with age. There was a decreasing trend in the relative abundance of the *Proteobacteria* phylum, *Klebsiella* genus, and *Streptococcus* genus with age in both boys and girls (all *P* trend < 0.05 for test of the trend with age). Conversely, there was an increasing trend in the relative abundance of the *Bacteroidetes* phylum with age (*P* trend = 0.046; Fig. 2C and D; Tables S1 and S2). There was a decreasing trend in the abundance of ABC transporter function and *oleC* in infants with age (all *P* trend < 0.05; Tables S3 and S4). Boys and girls delivered by cesarean section exhibited similar features in their gut microbiota (Fig. 2; Fig. S2).

### Mode of delivery and gut microbiota in the first 6 months of life

**Fig 3:**
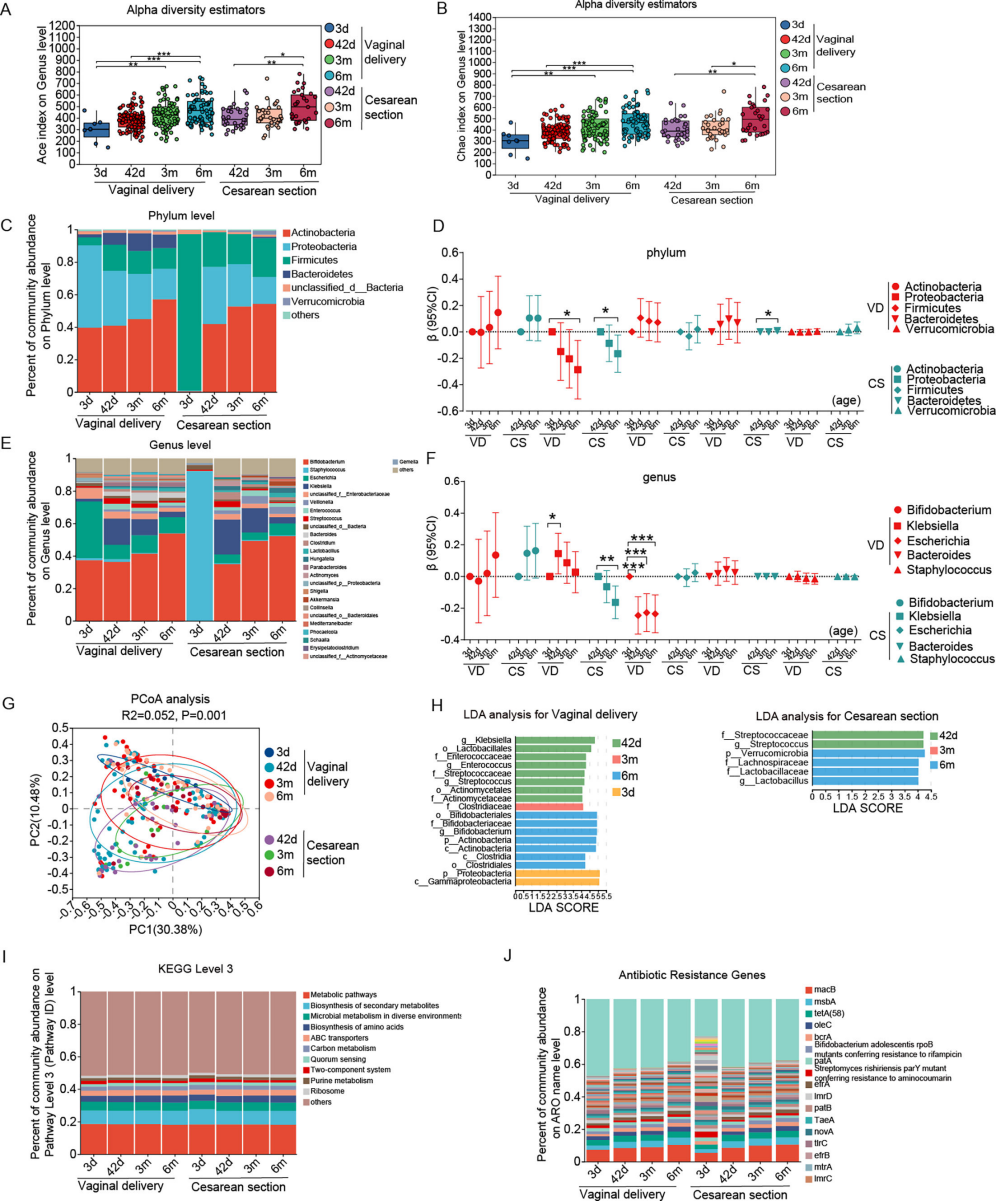
The alpha diversity on ACE index (**A**), Chao index (**B**), composition of phylum (**C and D**) and genus (**E and F**), β diversity (**G**), LDA (**H**), functional composition at KEGG level 3 (**I**), and antibiotics resistance genes (**J**) of gut microbiota from birth to 6 months of age between infants born by vaginal delivery and those by cesarean section. Note: 3d, age 3 days; 42d, age 42 days; 3m, age 3 months; 6m, age 6 months. VD, vaginal delivery; CS, cesarean section. *P < 0.05, **P < 0.01, and ***P < 0.001.

**Fig 4:**
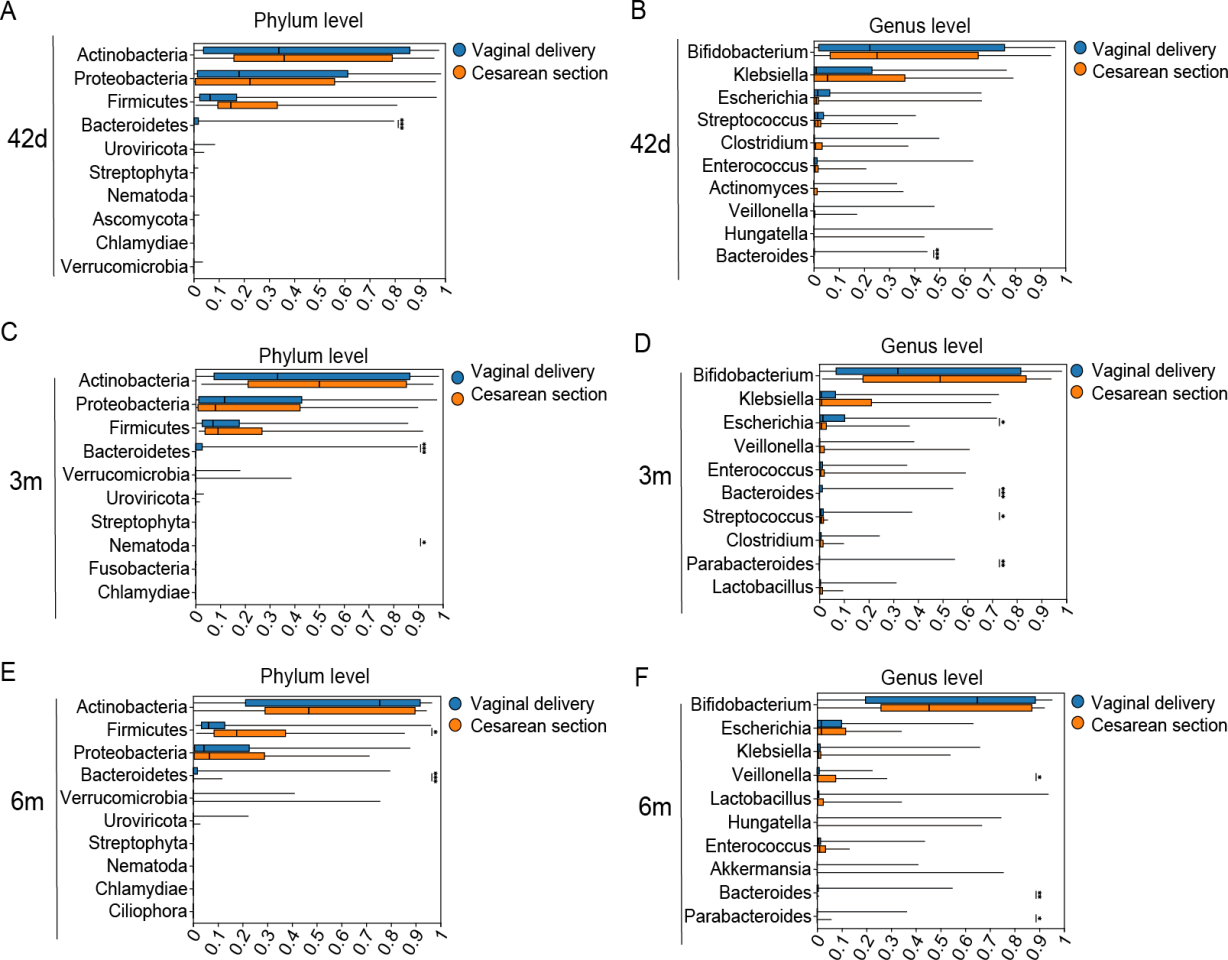
Differential microbial communities in phylum (**A, C, and E**) and genus (**B, D, and F**) between infants born by vaginal delivery and those by cesarean section at age 42 days, 3 months, and 6 months. Note: 42d, age 42 days; 3m, age 3 months; 6m, age 6 months. *P < 0.05, **P < 0.01, and ***P < 0.001 for Welch’s t test.

As expected, there were significant differences in gut microbiota between infants born by cesarean section and vaginal delivery (Fig. 3 and 4; Tables S1 through S4). Infants born by cesarean section exhibited a similar gut microbiota developmental trajectory, but with a slower increase in alpha diversity over time, compared with vaginally delivered infants (Fig. 3A and B; Fig. S3A through D), while there was no significant difference in alpha diversity between infants born by vaginal delivery and those born by cesarean section at each age point (Fig. 3A and B; Fig. S3A through D). We conducted PCoA and observed that the gut microbiota in infants born by cesarean section appeared to cluster separately from those in vaginally delivered infants at age 3 months and younger (Fig. 3G). As for composition, infants born by cesarean section consistently exhibited lower abundance of *Bacteroidetes* phylum in all ages: 8% lower at age 42 days (95% CI: 1%, 15%), 12% lower at 3 months (95% CI: 3%, 21%), and 8% lower at 6 months (95% CI: 1%, 16%). Similarly, the relative abundance of *Bacteroides* genus was also lower in infants born by cesarean section at 42 days, 3 months, and 6 months (Fig. 4; Tables S1 and S2). Moreover, cesarean-delivered infants exhibited a 0.02% lower abundance of the *Nematoda* phylum (mean ± SD: 0.06% ± 0.03% vs. 0.08% ± 0.05%, *P* = 0.04), 5% lower *Escherichia* genus (4% ± 9% vs. 10% ± 17%, *P* = 0.03), 3% lower *Parabacteroides* genus (0.01% ± 0.005% vs. 3% ± 9%, *P* = 0.008), and 2% lower *Streptococcus* genus (1% ± 1% vs. 3% ± 6%, *P* = 0.03) at age 3 months. At age 6 months, they showed 11% higher relative abundances of the *Firmicutes* phylum (24% ± 21% vs. 13% ± 18%, *P* = 0.02) and 3% higher in *Veillonella* genus (4% ± 6% vs. 1% ± 4%, *P* = 0.02), but 2% lower *Parabacteroides* genus (0.2% ± 1% vs. 3% ± 8%, *P* = 0.01) (Fig. 4) than those vaginal-delivered infants. With age going, infants born by vaginal delivery and cesarean section had similar decreasing trends in the relative abundance of *Proteobacteria* phylum and *Klebsiella* genus (Fig. 3D and F; Table S1 and S2). In contrast, the functional composition of gut microbiota at KEGG level 3 was similar between infants born by cesarean section and those delivered vaginally (Fig. 3I and J; Tables S3 and S4).

### ART treatments and infant gut microbiota

**Fig 5:**
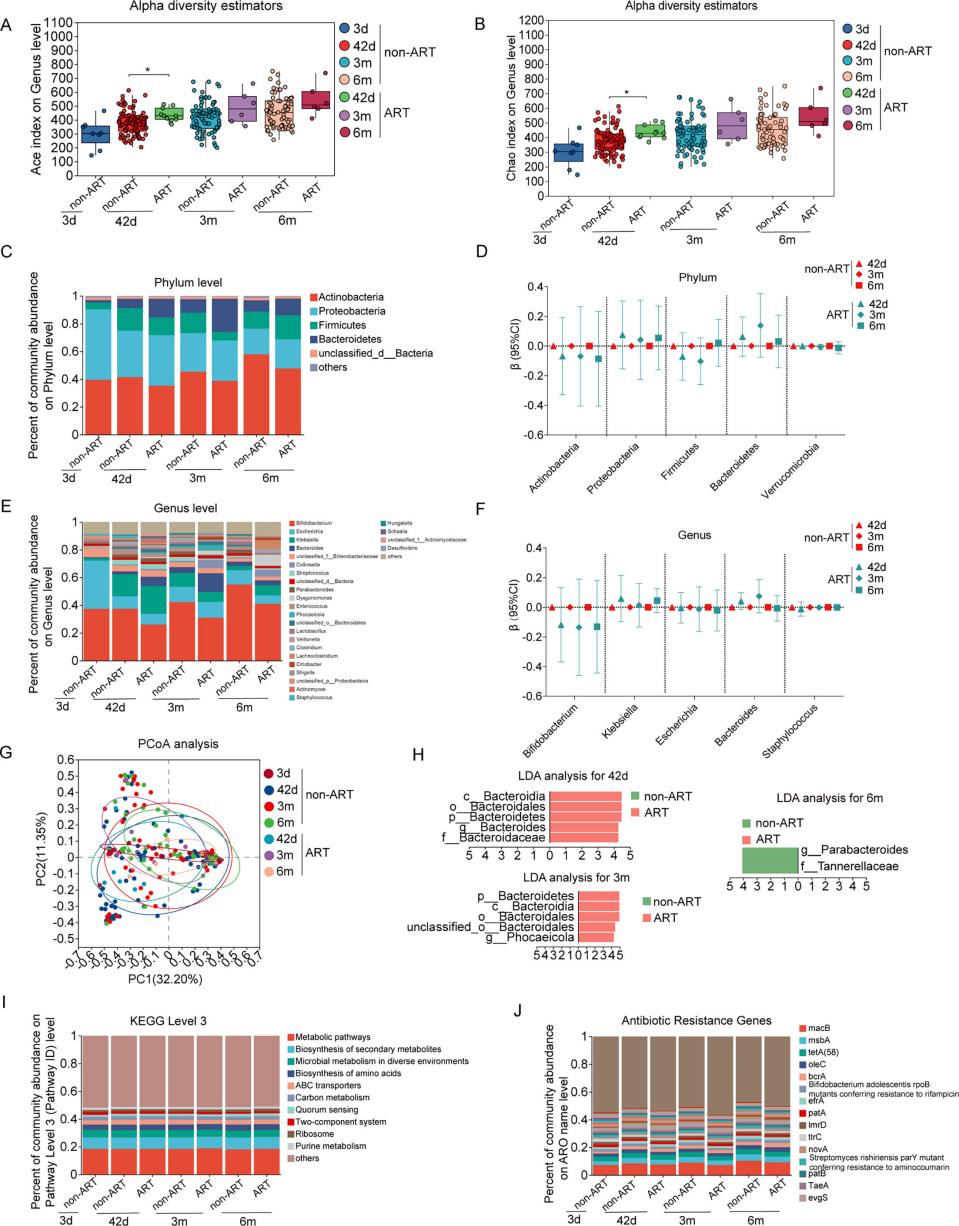
The alpha diversity on ACE index (**A**), Chao index (**B**), composition of phylum (**C and D**) and genus (**E and F**), β diversity (**G**), LDA (**H**), functional composition at KEGG level 3 (**I**), and antibiotics resistance genes (**J**) of gut microbiota from birth to 6 months of age between ART and non-ART infants born by vaginal delivery. Note: 3d, age 3 days; 42d, age 42 days; 3m, age 3 months; 6m, age 6 months. *P < 0.05.

We further explored the association between ART treatments and gut microbiota. Among infants born by vaginal delivery, compared with non-ART infants, infants conceived by ART had slightly higher alpha diversity indexes: 58.5 higher ACE (*P *= 0.004; Fig. 5A), 58.5 higher Chao (*P *= 0.004; Fig. 5B), 58.5 higher Sobs (*P *= 0.004; Fig. S4D), 0.55 higher Shannon (*P *= 0.02; Fig. S4C), 0.09 higher Pielou’s evenness (*P *= 0.02; Fig. S4B), and 0.19 lower Simpson index (*P *= 0.02; Fig. S4A) at 42 days of age (Fig. 5A and B; Fig. S4A through D), indicating an increase in richness and evenness in ART infants. Moreover, LEfSe analysis showed that gut microbiota of ART infants were more enriched in the *Bacteroidetes* phylum both at 42 days and 3 months of age (Fig. 5H), but the difference were not statistically significant (Fig. 5D; Table S5). The PCoA showed that non-ART infants and ART infants had similar clusters in their gut microbiota (Fig. 5G). Non-ART infants and ART infants had similar composition at KEGG level 3 and ARGs in gut microbiota (Fig. 5I and J; Tables S7 and S8).

**Fig 6:**
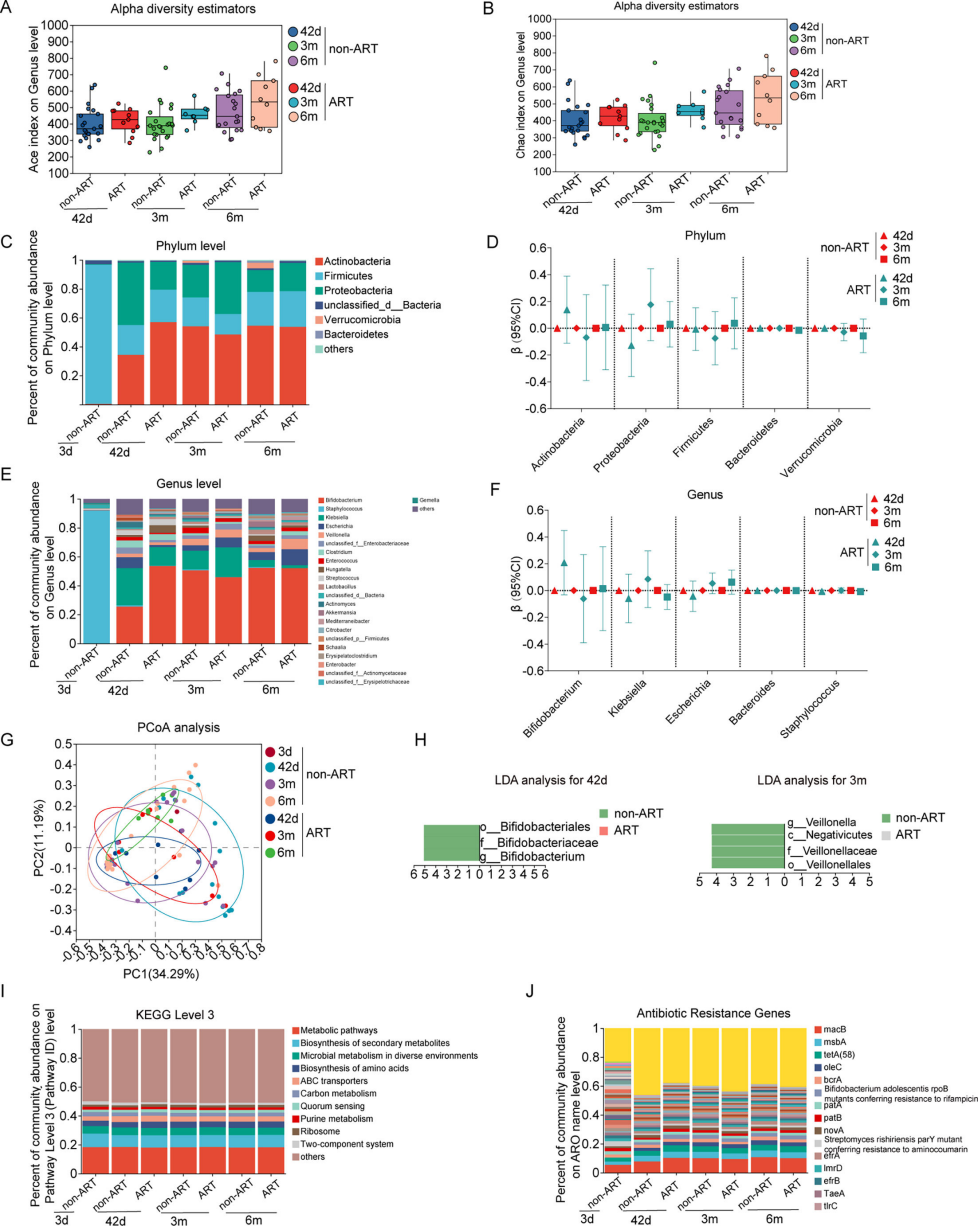
The alpha diversity on ACE index (**A**), Chao index (**B**), composition of phylum (**C and D**) and genus (**E and F**), β diversity (**G**), LDA (**H**), functional composition at KEGG level 3 (**I**), and antibiotic resistance genes (**J**) of gut microbiota from birth to 6 months of age between ART and non-ART infants born by cesarean section. Note: 3d, age 3 days; 42d, age 42 days; 3m, age 3 months; 6m, age 6 months. *P < 0.05.

Among infants born by cesarean section, the alpha diversity (Fig. 6A and B; Fig. S5A through D), taxonomic (Fig. 6C through H; Table S5 and S6), functional composition (Fig. 6I; Table S7) and ARGs (Fig. 6J; Table S8) were similar between ART and non-ART infants, but the abundance of *Bifidobacterium* genus was slightly higher in the ART infants at age 42 days (*P*=0.09; Fig. 6F; Table S6).

## DISCUSSION

This prospective study characterized gut microbiota development and antibiotic resistome dynamics in infants during the first 6 months of life. We observed age-dependent increases in microbial diversity and shifts in ARGs and metabolic pathways dominating KEGG level 3 functions across all ages. Boys and girls had similar features. Compared with vaginally delivered infants, cesarean-delivered infants showed a shallower alpha diversity trajectory and lower abundance of *Bacteroidetes* phylum and *Bacteroides* genus at each age point. Compared with non-ART infants, ART infants had slightly higher alpha diversity (ACE/Chao/Sobs/Shannon/Pielou’s evenness) at age 42 days among infants born by vaginal delivery.

In this study, we profiled the evolution of the gut microbiota and antibiotic resistome during the first 6 months of life, characterized by increasing alpha diversity with age, which was consistent with previous studies reporting increasing alpha diversity and decreasing beta diversity in the first year of postnatal life (18). In our study, the infant gut microbiota was predominated by *Actinobacteria*, *Proteobacteria*, *Firmicutes*, and *Bacteroidetes* phyla and *Bifidobacterium*, *Escherichia*, and *Klebsiella* genera—in line with the findings among infants from Japan (19), the United States (20, 21), and Europe (10). This might be explained by a predominantly aerobic environment at birth (2). Thereafter, the gut gradually becomes anaerobic, thereby allowing the colonization of anaerobes such as *Bifidobacterium* (*Actinobacteria* phylum), *Clostridium* (*Firmicutes* phylum), and *Bacteroides* (*Bacteroidetes* phylum) (22). Previous studies showed higher levels of specific ARGs in the gut microbiome of infants compared to adults, even when not exposed to antibiotics. Furthermore, higher relative abundances of ARGs were observed in younger infants compared to older children in other studies (10). Delivery mode, breastfeeding type, and antibiotic exposure may influence the ARG load in infants (23). Thus, the development of the infant’s resistome is linked to the evolution of the gut microbial composition.

We observed no association between infant sex and gut microbiota in early life, consistent with the findings in some studies (24, 25). Other studies reported a higher alpha diversity in female infants (26) or greater beta diversity in male infants (27). Another study did not find a significant effect of gender on the diversity and composition of the intestinal flora in infants but observed significant differences at the taxa level, with a higher relative abundance of *Erysipelotrichaceae*_UCG-003 and *Anaerostipes* in males, but a higher abundance of *Ruminiclostridium*, *Eubacterium*, *Senegalimassilia,* and *Senegalimassilia* gena*,* or *Firmicutes* and *Proteobacteria* phyla in females (27, 28). The sex differences in gut microbiota are usually attributed to hormonal differences between male and female hosts (29). This suggests that sex might be a surrogate factor for the influence of hormones on the colonization and composition of intestinal microbiota during the first 6 months of life.

Our study confirmed that infant gut microbiota was impacted by mode of delivery. Cesarean section disrupts the natural colonization process at birth, leading to a distinct microbial profile characterized by lower abundances of *Bacteroidetes* phylum and *Bacteroides* genus at 42 days, 3 months, and 6 months, which was consistent with the findings in previous studies (14), and some studies have even observed that children born by cesarean section lack *Bacteroides* species until 6–18 months of age (30), and a relatively lower gut microbial diversity at 2 years of age (31). There were inconsistencies remaining in the effects of delivery mode on early gut microbiota composition. In our study, infants born by cesarean section exhibited a similar trajectory but with a shallower slope in richness of gut microbiota, consistent with previous findings (32). However, some studies reported increased phylogenetic diversity, richness, and evenness in the meconium microbiota in cesarean-delivered infants (20). While others found no significant association between delivery mode and alpha diversity (21). During the first year of life, the beta diversity in vaginally delivered infants was greater than that in cesarean section-delivered infants (27). One hypothesis is that alteration of the microbiota in infants born by cesarean section stems from lack of exposure to the maternal vaginal microbiota. However, some studies found evidence for mother-to-child transmission of rectal rather than vaginal strains (30). Taken together, these results highlight an important role of vaginal delivery in structuring the infant gut microbiota in early life.

To the best of our knowledge, we profiled, for the first time, the gut microbiota in infants conceived after ART across the first 6 months after birth. We observed that ART conception was associated with increased richness and evenness of microbiota, as well as higher *Bacteroidetes* phylum and *Bacteroides* genus in infants born by vaginal delivery, and slightly higher *Bifidobacterium* genus in infants born by cesarean section. There was only one previous study that had explored the association between ART treatments and gut microbiota in the first 4 days after birth (16). They reported that ART was associated with reduced alpha diversity, lower relative abundance of *Bacteroidetes* phylum in neonatal gut microbiota (16). The difference between the studies may be due to sample size of ART infants and no stratified analysis on the mode of delivery. The ART treatments appeared to be associated with shifts in gut microbiota structure in early life. As for the underlying mechanism of these findings, we speculated that progesterone (33) and estrogen (34) used during ART treatment may change fetal intrinsic host microenvironment, which in turn may have a potential impact on microbiota maturation in later life. There were studies showing that reproductive hormones can mediate changes in the maternal gut microbiome during pregnancy and lactation (35).

This study described the dynamic changes in the gut microbiota during the first 6 months of life and explored the influence of key factors such as delivery mode, sex, and ART on microbiota establishment. These foundational data provided an important reference for future mechanistic research and potential implications for guiding clinical practice (e.g., determining optimal timing for microbiota interventions in cesarean-delivered infants), thus informing public health policy. The strength of this study was the prospective and standardized collection and handling of fecal samples, which reduced the risk of pre-analytical errors. The main limitation is the relatively small study sample size, especially at 3 days of age, because of low detection in microbiota.

### Conclusions

This longitudinal prospective study tracked the development of gut microbiota and their antibiotic resistome in Chinese infants during the first 6 months of life, revealing increasing diversity and shifts in dominant taxa and ARGs. ART treatments and mode of delivery may influence the diversity and composition of gut microbiota in early infancy.

## Data Availability

The data supporting the findings of this study are available within the article and its supplemental information files. The metagenomic raw sequencing data generated in this study have been deposited in the China National Center for Bioinformation (CNCB) under accession number CRA026837.

## SUPPLEMENTAL MATERIAL

Text S1 msystems.00502-25-s0001.docx Additional methods.

Supplemental material msystems.00502-25-s0002.docx Figures S1-S5; Tables S1-S8.

Checklist msystems.00502-25-s0003.docx The STORMS checklist.

ASM does not own the copyrights to Supplemental Material that may be linked to, or accessed through, an article. The authors have granted ASM a non-exclusive, world-wide license to publish the Supplemental Material files. Please contact the corresponding author directly for reuse.
