# Supplementary material for: https://webvpn.shsmu.edu.cn/https/77726476706e69737468656265737421e0e243912234265e7d0a80e296592e7bb7d62ae2c192eb/31091181/Gut microbiota development, antibiotic resistome, and related perinatal factors in early infancy
Source: mSystems. 2025 Jul 31;10(8):e00502-25. doi: 10.1128/msystems.00502-25 (PMC12363204; doi:10.1128/msystems.00502-25)
Supplement: Text S1 — Additional methods. [file msystems.00502-25-s0001.docx]

**DNA extraction, library construction, and metagenomic sequencing**

Total genomic DNA was extracted from fecal samples using the QIAamp PowerFecal Pro DNA Kit (QIAGEN, 51804) according to manufacturer’s instructions. Concentration and purity of extracted DNA were determined with TBS-380 and NanoDrop2000, respectively. DNA extract quality was checked on 1% agarose gel.

DNA extract was fragmented to an average size of about 400 bp using Covaris M220 (Gene Company Limited, China) for paired-end library construction. Paired-end library was constructed using NEXTflex^TM^ Rapid DNA-Seq (Bioo Scientific, Austin, TX, USA). Adapters containing the full complement of sequencing primer hybridization sites were ligated to the blunt-end of fragments. Paired-end sequencing was performed on Illumina Hiseq Xten (Illumina Inc., San Diego, CA, USA) at Majorbio Bio-Pharm Technology Co., Ltd. (Shanghai, China) using HiSeq X Reagent Kits according to the manufacturer’s instructions (www.illumina.com).

**Sequence quality control and genome assembly**

The raw reads from metagenome sequencing were used to generate clean reads by removing adaptor sequences, trimming and removing low-quality reads (reads with N bases, a minimum length threshold of 50bp and a minimum quality threshold of 20bp using the fastp (https://github.com/OpenGene/fastp, version 0.20.0) on the free online platform of Majorbio Cloud Platform (cloud.majorbio.com). The clean reads were mapped to the human hg38 reference genome using BWA (http://bio-bwa.sourceforge.net, version 0.7.9a) to identify and remove the human host originated reads. These high-quality reads were then assembled to contigs using MEGAHIT (parameters: kmer_min=4, kmer_max=97, step=10) (<https://github.com/voutcn/megahit>, version 1.1.2) which generates succinct de Bruijn graphs. Contigs with the length at or over 300 bp were selected as the final assembling result.

**Gene prediction, taxonomy, and functional annotation**

Open reading frames (ORFs) in contigs were identified using MetaGene (http://metagene.cb.k.u-tokyo.ac.jp/). The predicted ORFs with length at $\geq$100 bp were retrieved and translated into amino acid sequences using the NCBI translation table (http://www.ncbi.nlm.nih.gov/Taxonomy/taxonomyhome.html/index.cgi?chapter=tgencodes#SG1.

A non-redundant gene catalog was constructed using CD-HIT (http://www.bioinformatics.org/cd-hit/, version 4.6.1) with 90% sequence identity and 90% coverage. Reads after quality control were mapped to the non-redundant gene catalog with 95% identity using SOAPaligner (http://soap.genomics.org.cn/, version 2.21), and gene abundance in each sample was evaluated.

[Representative sequences](http://en.wikipedia.org/wiki/Representative_sequences) of non-redundant gene catalog were annotated based on the NCBI NR database using blastp as implemented in DIAMOND v0.9.19 with e-value cutoff of 1e^-5^ using Diamond (<https://github.com/bbuchfink/diamond/>, v0.9.19) for taxonomic annotations. The KEGG annotation was conducted using Diamond (<https://github.com/bbuchfink/diamond/>, v0.9.19) against the Kyoto Encyclopedia of Genes and Genomes database (http://www.kegg.jp/, version 94.2) with an e-value cutoff of 1e^-5^.

Linear discriminant analysis effect size (LEfSe analysis) ^[1]^ based on the non-parametric factorial Kruskal–Wallis (KW) sum-rank test was used to further analyze the significantly different taxon abundance between specified groups.

For counting the number of phylum, genus and ARGs, species with relative abundance <0.01 were combined.

Reference

1. Segata N, Izard J, Waldron L*, et al.* Metagenomic biomarker discovery and explanation. **Genome Biol** **2011**, 12(6)**:** R60.
