# Supplementary material for: https://webvpn.shsmu.edu.cn/https/77726476706e69737468656265737421e0e243912234265e7d0a80e296592e7bb7d62ae2c192eb/31091181/Gut microbiota development, antibiotic resistome, and related perinatal factors in early infancy
Source: mSystems. 2025 Jul 31;10(8):e00502-25. doi: 10.1128/msystems.00502-25 (PMC12363204; doi:10.1128/msystems.00502-25)
Supplement: Supplemental material — Figures S1-S5; Tables S1-S8. [file msystems.00502-25-s0002.docx]

**Supplementary table 1.** Relative abundance of the top 6 dominant gut microbiota at phylum level in infants by mode of delivery

| **Mode of delivery** | **Phylum** | **Actinobacteria** |  | **Proteobacteria** | | **Firmicutes** | |
| --- | --- | --- | --- | --- | --- | --- | --- |
|  | Age | Mean$\pm$sd | Adjusted $\beta$(95%CI) | Mean$\pm$sd | Adjusted $\beta$(95%CI) | Mean$\pm$sd | Adjusted $\beta$(95%CI) |
| Vaginal delivery | 3d (n=8) | 0.43±0.37 | ref | 0.48±0.36 | ref | 0.05±0.05 | ref |
|  | 42d (n=104) | 0.42±0.38 | -0.004(-0.28,0.27) | 0.31±0.34 | -0.15(-0.37,0.07) | 0.17±0.24 | 0.11(-0.04,0.25) |
|  | 3m (n=77) | 0.46±0.38 | 0.03(-0.24,0.31) | 0.25±0.30 | -0.20(-0.43,0.02) | 0.14±0.18 | 0.08(-0.07,0.23) |
|  | 6m (n=76) | 0.57±0.36 | 0.15(-0.13,0.42) | 0.17±0.25 | **-0.29(-0.51,-0.06)*** | 0.13±0.18 | 0.07(-0.08,0.22) |
|  | P-trend |  | **0.011** |  | **0.0006** |  | 0.531 |
| Sex | boy | - | ref | - | ref | **-** | ref |
|  | girl | - | -0.004(-0.09,0.10) | - | **-0.08(-0.15,-0.004)*** | **-** | 0.04(-0.01,0.09) |
| Cesarean section | 3d (n=1) | 0.01 |  | 0.004 |  | 0.96 |  |
|  | 42d (n=32) | 0.44±0.34 | ref | 0.32±0.34 | ref | 0.22±0.20 | ref |
|  | 3m (n=31) | 0.54±0.33 | 0.11(-0.06,0.28) | 0.25±0.29 | -0.09(-0.23,0.05) | 0.19±0.21 | -0.04(-0.14,0.07) |
|  | 6m (n=29) | 0.54±0.34 | 0.11(-0.07,0.28) | 0.16±0.19 | **-0.17(-0.31,-0.03)*** | 0.24±0.21 | 0.02(-0.09,0.12) **^#^** |
|  | P-trend |  | 0.214 |  | **0.019** |  | 0.730 |
| Sex | boy | - | ref | - | ref | **-** | ref |
|  | girl | - | 0.07(-0.08,0.22) | - | -0.10(-0.22,0.02) | **-** | 0.001(-0.09,0.09) |
|  |  | **Bacteroidetes** |  | **Verrucomicrobia** |  | **Uroviricota** | |
|  | Age | Mean$\pm$sd | Adjusted $\beta$(95%CI) | Mean$\pm$sd | Adjusted $\beta$(95%CI) | Mean$\pm$sd | Adjusted $\beta$(95%CI) |
| Vaginal delivery | 3d (n=8) | 0.02±0.04 | ref | 0.002±0.01 | ref | 0.001±0.002 | ref |
|  | 42d (n=104) | 0.08±0.19 | 0.06(-0.10,0.21) | 0.0004±0.003 | -0.003(-0.02,0.02) | 0.001±0.01 | 0.00002(-0.01,0.01) |
|  | 3m (n=77) | 0.12±0.24 | 0.10(-0.06,0.25) | 0.003±0.02 | -0.0005(-0.02,0.02) | 0.001±0.004 | -0.0003(-0.01,0.01) |
|  | 6m (n=76) | 0.09±0.20 | 0.07(-0.09,0.22) | 0.01±0.05 | 0.003(-0.02,0.02) | 0.003±0.03 | 0.002(-0.01,0.01) |
|  | P-trend |  | 0.438 |  | 0.181 |  | 0.384 |
| Sex | boy | - | ref | - | ref | **-** | ref |
|  | girl | - | 0.02(-0.03,0.07) | - | 0.005(-0.002,0.01) | **-** | 0.002(-0.01,0.01) |
| Cesarean section | 3d (n=1) | 0.00003 |  | 0.000002 |  | 0.001 |  |
|  | 42d (n=32) | 0.001±0.0005 | ref**^#^** | 0.00003±0.00004 | ref | 0.002±0.01 | ref |
|  | 3m (n=31) | 0.001±0.001 | 0.0005(-0.01,0.01)**^#^** | 0.01±0.07 | 0.02(-0.03,0.06) | 0.001±0.03 | -0.001(-0.004,0.002) |
|  | 6m (n=29) | 0.01±0.03 | **0.01(0.0003,0.02)*^#^** | 0.03±0.14 | 0.03(-0.02,0.08) | 0.002±0.01 | 0.0002(-0.003,0.003) |
|  | P-trend |  | **0.046** |  | 0.187 |  | 0.901 |
| Sex | boy | - | ref | - | ref | **-** | ref |
|  | girl | - | 0.004(-0.003,0.01) | - | 0.02(-0.01,0.06) | **-** | 0.001(-0.002,0.003) |

All regression models included infant age, sex (female, male) and feeding pattern (exclusive breast-feeding, mixed feeding) as covariates in the same models. * p<0.05 for generalized linear regression between gut microbiota and age. # p<0.05 for generalized linear regression between gut microbiota and mode of delivery. The bolded data double-indicated the statistically significant p < 0.05.

**Supplementary table 2.** Relative abundance of the top 6 dominant gut microbiota at genus level in infants by mode of delivery

| **Mode of delivery** | **Genus** | **Bifidobacterium** |  | **Klebsiella** | | **Escherichia** |  |
| --- | --- | --- | --- | --- | --- | --- | --- |
|  | Age | Mean$\pm$sd | Adjusted $\beta$(95%CI) | Mean$\pm$sd | Adjusted $\beta$(95%CI) | Mean$\pm$sd | Adjusted $\beta$(95%CI) |
| Vaginal delivery | 3d (n=8) | 0.40±0.37 | ref | 0.02±0.03 | ref | 0.33±0.26 | ref |
|  | 42d (n=104) | 0.38±0.37 | -0.03(-0.29,0.23) | 0.15±0.23 | **0.15(0.02,0.27)*** | 0.08±0.15 | **-0.25(-0.37,-0.13)***** |
|  | 3m (n=77) | 0.43±0.37 | 0.02(-0.25,0.29) | 0.09±0.17 | 0.09(-0.04,0.22) | 0.10±0.17 | **-0.23(-0.35,-0.11)***** |
|  | 6m (n=76) | 0.54±0.36 | 0.13(-0.13,0.40) | 0.03±0.09 | 0.03(-0.10,0.16) | 0.09±0.16 | **-0.24(-0.36,-0.12)***** |
|  | P-trend |  | **0.005** |  | **0.0005** |  | 0.297 |
| Sex | boy | - | ref | - | ref | **-** | ref |
|  | girl | - | 0.01(-0.08,0.09) | - | **-0.07(-0.12,-0.03)**** | **-** | 0.02(-0.02,0.06) |
| Cesarean section | 3d (n=1) | 0.0001 |  | 0.0001 |  | 0.00005 |  |
|  | 42d (n=32) | 0.36±0.34 | ref | 0.20±0.25 | ref | 0.05±0.15 | ref |
|  | 3m (n=31) | 0.50±0.34 | 0.15(-0.02,0.33) | 0.14±0.22 | -0.07(-0.17,0.04) | 0.04±0.09 | -0.012(-0.07,0.04) |
|  | 6m (n=29) | 0.52±0.34 | 0.17(-0.01,0.34) | 0.04±0.10 | **-0.16(-0.27,-0.06)**** | 0.07±0.10 | 0.02(-0.03,0.08) |
|  | P-trend |  | 0.058 |  | **0.002** |  | 0.454 |
| Sex | boy | - | ref | - | ref | **-** | ref |
|  | girl | - | 0.03(-0.12,0.18) | - | -0.03(-0.12,0.06) | **-** | **-0.05(-0.10,-0.01)*** |
|  |  | **Bacteroides** |  | **Staphylococcus** |  | **Streptococcus** | |
|  | Age | Mean$\pm$sd | Adjusted $\beta$(95%CI) | Mean$\pm$sd | Adjusted $\beta$(95%CI) | Mean$\pm$sd | Adjusted $\beta$(95%CI) |
| Vaginal delivery | 3d (n=8) | 0.01±0.02 | ref | 0.01±0.03 | ref | 0.01$\pm$0.02 | ref |
|  | 42d (n=104) | 0.03±0.08 | 0.02(-0.06,0.09) | 0.02±0.07 | -0.0001(-0.03,0.03) | 0.03$\pm$0.06 | 0.01(-0.01,0.06) |
|  | 3m (n=77) | 0.05±0.13 | 0.04(-0.03,0.12) | 0.004±0.01 | -0.01(-0.05,0.02) | 0.03$\pm$0.06 | 0.02(-0.02,0.05) |
|  | 6m (n=76) | 0.03±0.10 | 0.02(-0.05,0.10) | 0.001±0.003 | -0.02(-0.05,0.02) | 0.01$\pm$0.02 | 0.001(-0.04,0.04) |
|  | P-trend |  | 0.418 |  | **0.017** |  | **0.010** |
| Sex | boy | - | ref | - | ref | **-** | ref |
|  | girl | - | 0.02(-0.005,0.05) | - | 0.01(-0.004,0.02) | **-** | -0.004(-0.02,0.01) |
| Cesarean section | 3d (n=1) | 0.00001 |  | 0.92 |  | 0.008 |  |
|  | 42d (n=32) | 0.0002±0.0002 | ref**^#^** | 0.01±0.01 | ref | 0.04±0.07 | ref |
|  | 3m (n=31) | 0.0003±0.0005 | 0.0001(-0.0004,0.001) **^#^** | 0.0003±0.0005 | -0.002(-0.01,0.01) | 0.01±0.01 | **-0.03(-0.05,-0.004)*** |
|  | 6m (n=29) | 0.001±0.002 | 0.0005(-0.00001,0.001) | 0.004±0.02 | -0.003(-0.01,0.01) | 0.01±0.01 | **-0.03(-0.05,-0.01)**** |
|  | P-trend |  | 0.057 |  | 0.557 |  | **0.006** |
| Sex | boy | - | ref | - | ref | **-** | ref |
|  | girl | - | 0.0001(-0.0002,0.001) | - | 0.005(-0.003,0.01) | **-** | -0.01(-0.03,0.01) |

All regression models included infant age, sex (female, male) and feeding pattern (exclusive breast-feeding, mixed feeding) as covariates in the same models. * p<0.05, ** p<0.01 and *** p<0.001 for generalized linear regression between gut microbiota and age. # p<0.05 for generalized linear regression between gut microbiota and mode of delivery. The bolded data double-indicated the statistically significant p < 0.05.

**Supplementary table 3.** Relative abundance of the functional composition of the gut microbiota at KEGG level 3 of gut microbiota in infants by mode of delivery

| **Mode of delivery** | **Function** | **Metabolic pathways** |  | **Biosynthesis of secondary metabolites** | | **Microbial metabolism in diverse environments** | |
| --- | --- | --- | --- | --- | --- | --- | --- |
|  | Age | Mean$\pm$sd | Adjusted $\beta$(95%CI) | Mean$\pm$sd | Adjusted $\beta$(95%CI) | Mean$\pm$sd | Adjusted $\beta$(95%CI) |
| Vaginal delivery | 3d (n=8) | 0.186±0.005 | ref | 0.083±0.006 | ref | 0.054±0.005 | ref |
|  | 42d (n=104) | 0.185±0.007 | -0.001(-0.01,0.004) | 0.083±0.007 | 0.0002(-0.004,0.005) | 0.052±0.006 | -0.002(-0.01,0.002) |
|  | 3m (n=77) | 0.186±0.008 | -0.001(-0.01,0.01) | 0.085±0.006 | 0.001(-0.003,0.01) | 0.051±0.005 | -0.003(-0.01,0.001) |
|  | 6m (n=76) | 0.183±0.008 | -0.004(-0.01,0.002) | 0.086±0.005 | 0.003(-0.002,0.01) | 0.049±0.004 | **-0.004(-0.01,-0.001)*** |
|  | P-trend |  | 0.061 |  | **0.009** |  | **0.001** |
| Sex | boy | - | ref | - | ref | **-** | ref |
|  | girl | - | 0.0003(-0.002,0.002) | - | 0.001(-0.0002,0.003) | **-** | **-0.001(-0.003,-0.0001)*** |
| Cesarean section | 3d (n=1) | 0.183 |  | 0.094 |  | 0.052 |  |
|  | 42d (n=32) | 0.184±0.005 | ref | 0.083±0.007 | ref | 0.052±0.006 | ref |
|  | 3m (n=31) | 0.183±0.006 | -0.001(-0.004,0.003) | 0.084±0.007 | 0.001(-0.002,0.004) | 0.051±0.005 | -0.001(-0.003,0.002) |
|  | 6m (n=29) | 0.182±0.007 | -0.002(-0.005,0.002) | 0.085±0.005 | 0.002(-0.001,0.01) | 0.05±0.004 | -0.002(-0.004,0.001) |
|  | P-trend |  | 0.341 |  | 0.199 |  | 0.138 |
| Sex | boy | - | ref | - | ref | **-** | ref |
|  | girl | - | -0.001(-0.003,0.002) | - | 0.002(-0.001,0.005) | - | -0.001(-0.004,0.001) |
|  |  | **Biosynthesis of amino acids** | | **ABC transporters** | | **Carbon metabolism** | |
|  | Age | Mean$\pm$sd | Adjusted $\beta$(95%CI) | Mean$\pm$sd | Adjusted $\beta$(95%CI) | Mean$\pm$sd | Adjusted $\beta$(95%CI) |
| Vaginal delivery | 3d (n=8) | 0.038±0.009 | ref | 0.030±0.008 | ref | 0.025±0.001 | ref |
|  | 42d (n=104) | 0.040±0.007 | 0.002(-0.003,0.01) | 0.035±0.009 | 0.004(-0.002,0.01) | 0.025±0.001 | -0.0001(-0.001,0.001) |
|  | 3m (n=77) | 0.041±0.007 | 0.003(-0.002,0.01) | 0.033±0.01 | 0.003(-0.004,0.01) | 0.025±0.002 | -0.0002(-0.001,0.001) |
|  | 6m (n=76) | 0.043±0.007 | 0.01(-0.0001,0.01) | 0.032±0.009 | 0.002(-0.005,0.01) | 0.025±0.002 | -0.001(-0.002,0.0005) |
|  | P-trend |  | **0.003** |  | 0.131 |  | **0.035** |
| Sex | boy | - | ref | - | ref | **-** | ref |
|  | girl | - | 0.001(-0.001,0.003) | - | 0.0003(-0.002,0.003) | **-** | 0.0001(-0.0002,0.0005) |
| Cesarean section | 3d (n=1) | 0.037 |  | 0.03 |  | 0.029 |  |
|  | 42d (n=32) | 0.04±0.007 | ref | 0.041±0.008 | ref**^#^** | 0.025±0.001 | ref |
|  | 3m (n=31) | 0.042±0.007 | 0.002(-0.001,0.01) | 0.036±0.008 | **-0.005(-0.01,-0.0005)*** | 0.025±0.001 | -0.0004(-0.001,0.0002) |
|  | 6m (n=29) | 0.043±0.005 | 0.003(-0.0001,0.01) | 0.036±0.01 | **-0.005(-0.01,-0.0003)* ^#^** | 0.025±0.001 | -0.001(-0.001,0.0001) |
|  | P-trend |  | 0.055 |  | **0.036** |  | 0.092 |
| Sex | boy | - | ref | - | ref | **-** | ref |
|  | girl | - | 0.002(-0.001,0.005) | - | -0.001(-0.005,0.003) | **-** | **-0.001(-0.001,-0.0002)**** |

All regression models included infant age, sex (female, male) and feeding pattern (exclusive breast-feeding, mixed feeding) as covariates in the same models. * p<0.05, ** p<0.01 for generalized linear regression between gut microbiota and age. # p<0.05 for generalized linear regression between gut microbiota and mode of delivery. The bolded data double-indicated the statistically significant p < 0.05.

**Supplementary table 4**. Antibiotic resistome of gut microbiota in infants from birth to age 6 months by mode of delivery

| **Mode of delivery** | **CARD** | **macB** | | **msbA** |  | **tetA(58)** |  |
| --- | --- | --- | --- | --- | --- | --- | --- |
|  | age | Mean$\pm$sd | $\beta$(95%CI) | Mean$\pm$sd | $\beta$(95%CI) | Mean$\pm$sd | $\beta$(95%CI) |
| Vaginal delivery | 3d (n=8) | 0.079±0.047 | ref | 0.026±0.011 | ref | 0.034±0.01 | ref |
|  | 42d (n=104) | 0.087±0.04 | 0.01(-0.02,0.04) | 0.038±0.018 | 0.01(-0.002,0.03) | 0.037±0.013 | 0.003(-0.01,0.01) |
|  | 3m (n=77) | 0.092±0.041 | 0.01(-0.02,0.04) | 0.04±0.018 | 0.01(-0.0004,0.03) | 0.035±0.013 | 0.001(-0.01,0.01) |
|  | 6m (n=76) | 0.104±0.041 | 0.03(-0.005,0.06) | 0.044±0.022 | **0.02(0.004,0.03)*** | 0.036±0.012 | 0.002(-0.01,0.01) |
|  | P-trend |  | **0.005** |  | **0.009** |  | 0.703 |
| Sex | boy | - | ref | - | ref | **-** | ref |
|  | girl | - | 0.002(-0.01,0.01) | - | 0.001(-0.003,0.01) | **-** | 0.002(-0.002,0.005) |
| Cesarean section | 3d (n=1) | 0.055 |  | 0.023 |  | 0.028 |  |
|  | 42d (n=32) | 0.089±0.037 | ref | 0.041±0.017 | ref | 0.039±0.014 | ref |
|  | 3m (n=31) | 0.102±0.039 | 0.01(-0.004,0.03) | 0.042±0.019 | 0.002(-0.01,0.01) | 0.039±0.012 | 0.001(-0.005,0.01) |
|  | 6m (n=29) | 0.106±0.036 | 0.02(-0.002,0.04) | 0.044±0.019 | 0.003(-0.01,0.01) | 0.04±0.011 | 0.002(-0.005,0.01) |
|  | P-trend |  | 0.07 |  | 0.482 |  | 0.592 |
| Sex | boy | - | ref | - | ref | **-** | ref |
|  | girl | - | 0.01(-0.001,0.03) | - | 0.001(-0.01,0.01) | **-** | 0.0001(-0.01,0.01) |
|  | age | **oleC** |  | **bcrA** |  | **bifidobacterium** | |
|  |  | Mean$\pm$sd | $\beta$(95%CI) | Mean$\pm$sd | $\beta$(95%CI) | Mean$\pm$sd | $\beta$(95%CI) |
| Vaginal delivery | 3d (n=8) | 0.023±0.009 | ref | 0.014±0.008 | ref | 0.02±0.02 | ref |
|  | 42d (n=104) | 0.025±0.012 | 0.002(-0.01,0.01) | 0.023±0.011 | **0.01(0.001,0.02)*** | 0.02±0.01 | 0.001(-0.01,0.01) |
|  | 3m (n=77) | 0.024±0.011 | 0.001(-0.01,0.01) | 0.023±0.009 | **0.01(0.001,0.02)*** | 0.02±0.01 | 0.002(-0.01,0.01) |
|  | 6m (n=76) | 0.03±0.013 | 0.01(-0.002,0.02) | 0.023±0.012 | **0.01(0.002,0.02)*** | 0.02±0.01 | 0.005(-0.01,0.02) |
|  | P-trend |  | **0.01** |  | 0.164 |  | 0.099 |
| Sex | boy | - | ref | - | ref | **-** | ref |
|  | girl | - | 0.001(-0.002,0.004) | - | 0.001(-0.002,0.003) | **-** | 0.001(-0.003,0.004) |
| Cesarean section | 3d (n=1) | 0.0002 |  | 0.021 |  | 0.0002 |  |
|  | 42d (n=32) | 0.022±0.011 | ref | 0.021±0.008 | ref | 0.02±0.02 | ref |
|  | 3m (n=31) | 0.028±0.012 | **0.006(0.0002,0.01)*** | 0.022±0.011 | 0.001(-0.004,0.01) | 0.03±0.01 | 0.003(-0.005,0.01) |
|  | 6m (n=29) | 0.03±0.012 | **0.008(0.002,0.01)**** | 0.024±0.01 | 0.003(-0.002,0.01) | 0.03±0.02 | 0.003(-0.01,0.01) |
|  | P-trend |  | **0.008** |  | 0.180 |  | 0.480 |
| Sex | boy | - | ref | - | ref | **-** | ref |
|  | girl | - | **0.01(0.0003,0.01)*** | - | 0.003(-0.001,0.01) | **-** | -0.003(-0.01,0.004) |

All regression models included infant age, sex (female, male) and feeding pattern (exclusive breast-feeding, mixed feeding) as covariates in the same models. * p<0.05, ** p<0.01 for generalized linear regression between gut microbiota and age. The bolded data double-indicated the statistically significant p < 0.05.

**Supplementary table 5.** Relative abundance of the top 6 dominant gut microbiota at phylum level between infants born by vaginal delivery and those by cesarean section

| **Age** | **Phylum** | **Actinobacteria** | | **Proteobacteria** | | **Firmicutes** | |
| --- | --- | --- | --- | --- | --- | --- | --- |
| **Vaginal delivery** | ART | Mean$\pm$sd | Adjusted $\beta$(95%CI) | Mean$\pm$sd | Adjusted $\beta$(95%CI) | Mean$\pm$sd | Adjusted $\beta$(95%CI) |
| 3 days | no (n=8） | 0.43±0.37 | - | 0.48±0.36 | - | 0.05±0.05 | - |
|  | yes (n=0） | - | - | - | - | - | - |
| 42 days | no (n=94） | 0.43±0.39 | ref | 0.31±0.34 | ref | 0.17±0.24 | ref |
|  | yes (n=10) | 0.37±0.33 | -0.07(-0.33,0.19) | 0.35±0.29 | 0.07(-0.15,0.30) | 0.13±0.13 | -0.07(-0.23,0.09) |
| 3 months | no (n=71） | 0.47±0.38 | ref | 0.25±0.30 | ref | 0.15±0.18 | ref |
|  | yes (n=6) | 0.40±0.38 | -0.07(-0.40,0.27) | 0.28±0.26 | 0.04(-0.22,0.31) | 0.06±0.07 | -0.10(-0.26,0.06) |
| 6 months | no (n=70） | 0.58±0.37 | ref | 0.17±0.25 | ref | 0.13±0.18 | ref |
|  | yes (n=6) | 0.49±0.33 | -0.09(-0.41,0.23) | 0.20±0.22 | 0.06(-0.16,0.27) | 0.18±0.23 | 0.02(-0.14,0.18) |
| **Cesarean section** | |  |  |  |  |  |  |
| 3 days | no (n=1） | 0.01 |  | 0.004 |  | 0.96 |  |
|  | yes (n=0） | - |  | - |  | - |  |
| 42 days | no (n=21） | 0.37±0.36 | ref | 0.39±0.39 | ref | 0.22±0.21 | ref |
|  | yes (n=11) | 0.57±0.29 | 0.14(-0.11,0.39) | 0.19±0.18 | -0.13(-0.36,0.10) | 0.23±0.17 | -0.01(-0.17,0.15) |
| 3 months | no (n=23） | 0.55±0.35 | ref | 0.21±0.27 | ref | 0.20±0.23 | ref |
|  | yes (n=8) | 0.50±0.30 | -0.07(-0.39,0.25) | 0.34±0.33 | 0.18(-0.09,0.44) | 0.14±0.09 | -0.07(-0.27,0.12) |
| 6 months | no (n=19） | 0.54±0.38 | ref | 0.14±0.21 | ref | 0.24±0.24 | ref |
|  | yes (n=10) | 0.54±0.26 | 0.01(-0.31,0.32) | 0.19±0.15 | 0.03(-0.14,0.20) | 0.25±0.14 | 0.04(-0.15,0.23) |
|  |  | **Bacteroidetes** | | **Verrucomicrobia** | | **Uroviricota** | |
| **Vaginal delivery** | ART | Mean$\pm$sd | Adjusted $\beta$(95%CI) | Mean$\pm$sd | Adjusted $\beta$(95%CI) | Mean$\pm$sd | Adjusted $\beta$(95%CI) |
| 3 days | no (n=8） | 0.02±0.04 | - | 0.002±0.01 | - | 0.001±0.002 |  |
|  | yes (n=0） | - | - | - | - |  |  |
| 42 days | no (n=94） | 0.07±0.20 | ref | 0.0004±0.004 | ref | 0.001±0.01 | ref |
|  | yes (n=10) | 0.13±0.13 | 0.06(-0.07,0.20) | 0.0001±0.0001 | -0.0004(-0.003,0.002) | 0.0004±0.0004 | -0.001(-0.01,0.005) |
| 3 months | no (n=71） | 0.11±0.24 | ref | 0.003±0.02 | ref | 0.001±0.004 | ref |
|  | yes (n=6) | 0.25±0.30 | 0.14(-0.08,0.35) | 0.0001±0.0002 | -0.01(-0.02,0.01) | 0.0002±0.0001 | -0.001(-0.005,0.002) |
| 6 months | no (n=70） | 0.09±0.21 | ref | 0.01±0.05 | ref | 0.004±0.03 | ref |
|  | yes (n=6) | 0.12±0.15 | 0.03(-0.15,0.21) | 0.0001±0.0001 | -0.01(-0.05,0.03) | 0.0003±0.0002 | -0.01(-0.03,0.02) |
| **Cesarean section** | |  |  |  |  |  |  |
| 3 days | no (n=1） | 0.00003 |  | 0.000002 |  | 0.001 |  |
|  | yes (n=0） | - |  | - |  |  |  |
| 42 days | no (n=21） | 0.001±0.0004 | ref | 0.00002±0.00004 | ref | 0.002±0.01 | ref |
|  | yes (n=11) | 0.001±0.0005 | 0.00004(-0.0003,0.0004) | 0.00005±0.00004 | 0.00002(-0.00001,0.00005) | 0.0004±0.001 | -0.001(-0.01,0.004) |
| 3 months | no (n=23） | 0.0005±0.001 | ref | 0.02±0.08 | ref | 0.001±0.004 | ref |
|  | yes (n=8) | 0.001±0.0004 | 0.0005(-0.00003,0.001) | 0.0003±0.0007 | -0.03(-0.09,0.04) | 0.0005±0.001 | -0.0003(-0.003,0.003) |
| 6 months | no (n=19） | 0.01±0.03 | ref | 0.04±0.17 | ref | 0.002±0.01 | ref |
|  | yes (n=10) | 0.001±0.0004 | -0.01(-0.04,0.01) | 0.0001±0.000 | -0.06(-0.18,0.07) | 0.002±0.005 | -0.001(-0.01,0.004) |

All regression models included infant age, sex (female, male) and feeding pattern (exclusive breast-feeding, mixed feeding) as covariates in the same models.

**Supplementary table 6.** Relative abundance of the top 6 dominant gut microbiota at genus level between infants born by vaginal delivery and those by cesarean section

| **Age** | **Genus** | **Bifidobacterium** | | **Klebsiella** | | **Escherichia** |  |
| --- | --- | --- | --- | --- | --- | --- | --- |
| **Vaginal delivery** | ART | Mean$\pm$sd | Adjusted $\beta$(95%CI) | Mean$\pm$sd | Adjusted $\beta$(95%CI) | Mean$\pm$sd | Adjusted $\beta$(95%CI) |
| 3 days | no (n=8） | 0.40±0.37 | - | 0.02±0.03 | - | 0.33±0.26 | - |
|  | yes (n=0） | - | - | - | - | - | - |
| 42 days | no (n=94） | 0.39±0.37 | ref | 0.14±0.24 | ref | 0.08±0.15 | ref |
|  | yes (n=10) | 0.27±0.30 | -0.12(-0.37,0.13) | 0.19±0.21 | 0.06(-0.10,0.21) | 0.07±0.13 | -0.004(-0.11,0.10) |
| 3 months | no (n=71） | 0.43±0.37 | ref | 0.09±0.18 | ref | 0.10±0.17 | ref |
|  | yes (n=6) | 0.31±0.32 | -0.14(-0.46,0.19) | 0.07±0.08 | 0.01(-0.13,0.16) | 0.11±0.13 | -0.01(-0.16,0.14) |
| 6 months | no (n=70） | 0.55±0.36 | ref | 0.03±0.09 | ref | 0.09±0.16 | ref |
|  | yes (n=6) | 0.41±0.28 | -0.13(-0.44,0.18) | 0.07±0.10 | 0.04(-0.03,0.13) | 0.06±0.07 | -0.02(-0.16,0.12) |
| **Cesarean section** | |  |  |  |  |  |  |
| 3 days | no (n=1） | 0.0001 |  | 0.0001 |  | 0.00005 |  |
|  | yes (n=0） | - |  | - |  | - |  |
| 42 days | no (n=21） | 0.27±0.32 | ref | 0.24±0.28 | ref | 0.07±0.18 | ref |
|  | yes (n=11) | 0.53±0.30 | 0.21(-0.03,0.45) | 0.13±0.15 | -0.06(-0.24,0.12) | 0.01±0.02 | -0.04(-0.16,0.07) |
| 3 months | no (n=23） | 0.51±0.36 | ref | 0.12±0.20 | ref | 0.04±0.09 | ref |
|  | yes (n=8) | 0.47±0.30 | -0.06(-0.39,0.27) | 0.19±0.28 | 0.08(-0.13,0.30) | 0.07±0.09 | 0.05(-0.02,0.13) |
| 6 months | no (n=19） | 0.52±0.38 | ref | 0.04±0.01 | ref | 0.05±0.10 | ref |
|  | yes (n=10) | 0.52±0.25 | 0.01(-0.30,0.33) | 0.02±0.03 | -0.05(-0.14,0.04) | 0.11±0.10 | 0.06(-0.03,0.15) |
|  |  | **Bacteroides** |  | **Staphylococcus** | | **Streptococcus** | |
| **Vaginal delivery** | ART | Mean$\pm$sd | Adjusted $\beta$(95%CI) | Mean$\pm$sd | Adjusted $\beta$(95%CI) | Mean$\pm$sd | Adjusted $\beta$(95%CI) |
| 3 days | no (n=8） | 0.01±0.02 | - | 0.02±0.03 | - | 0.01±0.02 |  |
|  | yes (n=0） | - | - | - | - |  |  |
| 42 days | no (n=94） | 0.02±0.08 | ref | 0.02±0.07 | ref | 0.03±0.06 | ref |
|  | yes (n=10) | 0.07±0.09 | 0.04(-0.02,0.10) | 0.004±0.01 | -0.01(-0.06,0.04) | 0.04±0.05 | -0.002(-0.04,0.04) |
| 3 months | no (n=71） | 0.05±0.12 | ref | 0.004±0.01 | ref | 0.03±0.06 | ref |
|  | yes (n=6) | 0.14±0.19 | 0.07(-0.04,0.19) | 0.002±0.003 | -0.002(-0.01,0.01) | 0.01±0.01 | -0.004(-0.06,0.05) |
| 6 months | no (n=70） | 0.04±0.10 | ref | 0.001±0.003 | ref | 0.01±0.02 | ref |
|  | yes (n=6) | 0.03±0.07 | -0.01(-0.09,0.08) | 0.0005±0.0003 | -0.0002(-0.003,0.003) | 0.01±0.02 | 0.0003(-0.01,0.02) |
| **Cesarean section** | |  |  |  |  |  |  |
| 3 days | no (n=1） | 0.00001 |  | - |  | 0.008 |  |
|  | yes (n=0） | - |  | - |  |  |  |
| 42 days | no (n=21） | 0.0002±0.0003 | ref | 0.01±0.02 | ref | 0.03±0.05 | ref |
|  | yes (n=11) | 0.0002±0.0002 | -0.00002(-0.0002,0.0002) | 0.002±0.002 | -0.01(-0.02,0.005) | 0.04±0.10 | 0.005(-0.05,0.06) |
| 3 months | no (n=23） | 0.0003±0.001 | ref | 0.01±0.02 | ref | 0.01±0.01 | ref |
|  | yes (n=8) | 0.0004±0.0004 | 0.0003(-0.0001,0.001) | 0.002±0.002 | 0.0003(-0.02,0.02) | 0.01±0.01 | -0.005(-0.01,0.004) |
| 6 months | no (n=19） | 0.001±0.002 | ref | 0.01±0.02 | ref | 0.01±0.02 | ref |
|  | yes (n=10) | 0.0003±0.0002 | -0.001(-0.002,0.001) | 0.0005±0.0003 | -0.01(-0.02,0.01) | 0.003±0.002 | -0.01(-0.02,0.01) |

All regression models included infant age, sex (female, male) and feeding pattern (exclusive breast-feeding, mixed feeding) as covariates in the same models.

**Supplementary table 7.** Relative abundance of the functional composition of the gut microbiota at KEGG level 3 between infants born by vaginal delivery and those by cesarean section

| **Age** | **Function** | **Metabolic pathways** | | **Biosynthesis of secondary metabolites** | | **Microbial metabolism in diverse environments** | | **Biosynthesis of amino acids** | | **ABC transporters** | | **Carbon metabolism** | |
| --- | --- | --- | --- | --- | --- | --- | --- | --- | --- | --- | --- | --- | --- |
| **Vaginal delivery** | ART | Mean$\pm$sd | Adjusted $\beta$(95%CI) | Mean$\pm$sd | Adjusted $\beta$(95%CI) | Mean$\pm$sd | Adjusted $\beta$(95%CI) | Mean$\pm$sd | Adjusted $\beta$(95%CI) | Mean$\pm$sd | Adjusted $\beta$(95%CI) | Mean$\pm$sd | Adjusted $\beta$(95%CI) |
| 3 days | no (n=8） | 0.186±0.005 | - | 0.083±0.006 | - | 0.054±0.005 | - | 0.038±0.009 | - | 0.03±0.008 | - | 0.025±0.001 | - |
|  | yes (n=0） | - | - | - | - | - | - | - | - | - | - | - | - |
| 42 days | no (n=94） | 0.185±0.007 | ref | 0.083±0.007 | ref | 0.052±0.006 | ref | 0.04±0.008 | ref | 0.034±0.009 | ref | 0.025±0.001 | ref |
|  | yes (n=10) | 0.186±0.006 | 0.001(-0.004,0.01) | 0.084±0.007 | -0.0001(-0.005,0.005) | 0.052±0.006 | 0.001(-0.003,0.01) | 0.039±0.006 | -0.002(-0.01,0.003) | 0.035±0.007 | 0.0003(-0.01,0.01) | 0.026±0.001 | 0.0003(-0.001,0.001) |
| 3 months | no (n=71） | 0.185±0.008 | ref | 0.084±0.006 | ref | 0.051±0.005 | ref | 0.041±0.008 | ref | 0.033±0.01 | ref | 0.025±0.002 | ref |
|  | yes (n=6) | 0.19±0.007 | 0.01(-0.002,0.01) | 0.085±0.004 | 0.0001(-0.01,0.01) | 0.051±0.005 | 0.001(-0.004,0.005) | 0.04±0.006 | -0.002(-0.01,0.005) | 0.028±0.007 | -0.006(-0.01,0.003) | 0.026±0.001 | 0.0007(-0.001,0.002) |
| 6 months | no (n=70） | 0.182±0.008 | ref | 0.086±0.005 | ref | 0.049±0.004 | ref | 0.043±0.007 | ref | 0.032±0.009 | ref | 0.025±0.002 | ref |
|  | yes (n=6) | 0.186±0.008 | 0.003(-0.004,0.01) | 0.086±0.004 | -0.0001(-0.005,0.005) | 0.05±0.005 | 0.0005(-0.003,0.004) | 0.043±0.006 | -0.001(-0.01,0.01) | 0.033±0.004 | 0.001(-0.01,0.01) | 0.025±0.002 | 0.001(-0.001,0.002) |
| **Cesarean section** |  |  |  |  |  |  |  |  |  |  |  |  |  |
| 3 days | no (n=1） | 0.18 | - | 0.09 | - | 0.05 | - | 0.04 | - | 0.03 | - | 0.03 | - |
|  | yes (n=0） | - | - | - | - | - | - | - | - | - | - | - | - |
| 42 days | no (n=21） | 0.185±0.005 | ref | 0.083±0.008 | ref | 0.052±0.007 | ref | 0.038±0.007 | ref | 0.04±0.007 | ref | 0.026±0.001 | ref |
|  | yes (n=11) | 0.181±0.005 | -0.003(-0.01,0.001) | 0.085±0.005 | 0.001(-0.004,0.01) | 0.05±0.004 | -0.001(-0.01,0.004) | 0.043±0.005 | 0.003(-0.002,0.01) | 0.041±0.009 | 0.002(-0.005,0.01) | 0.025±0.001 | -0.001(-0.001,0.0002) |
| 3 months | no (n=23） | 0.183±0.007 | ref | 0.085±0.007 | ref | 0.051±0.005 | ref | 0.042±0.007 | ref | 0.036±0.008 | ref | 0.025±0.002 | ref |
|  | yes (n=8) | 0.185±0.004 | 0.003(-0.003,0.01) | 0.083±0.006 | -0.002(-0.01,0.005) | 0.053±0.005 | 0.004(-0.001,0.01) | 0.04±0.007 | -0.002(-0.01,0.005) | 0.035±0.006 | -0.002(-0.01,0.01) | 0.025±0.001 | 0.0001(-0.001,0.001) |
| 6 months | no (n=19） | 0.182±0.007 | ref | 0.085±0.006 | ref | 0.05±0.004 | ref | 0.043±0.006 | ref | 0.036±0.012 | ref | 0.025±0.001 | ref |
|  | yes (n=10) | 0.183±0.005 | 0.001(-0.01,0.01) | 0.085±0.003 | -0.0004(-0.005,0.004) | 0.05±0.003 | 0.0005(-0.003,0.004) | 0.043±0.004 | -0.0005(-0.01,0.004) | 0.036±0.005 | 0.002(-0.01,0.01) | 0.025±0.001 | 0.001(-0.001,0.002) |

All regression models included infant age, sex (female, male) and feeding pattern (exclusive breast-feeding, mixed feeding) as covariates in the same models.

**Supplementary table s8**. Antibiotic resistance of gut microbiota in infants from birth to age 6 months between infants born by vaginal delivery and those by cesarean section

| **Age** | **CARD** | **macB** | | **msbA** | | **tetA58** | | **oleC** | | **bcrA** | | **Bifidobacterium** | |
| --- | --- | --- | --- | --- | --- | --- | --- | --- | --- | --- | --- | --- | --- |
| **Vaginal delivery** | ART | Mean$\pm$sd | Adjusted $\beta$(95%CI) | Mean$\pm$sd | Adjusted $\beta$(95%CI) | Mean$\pm$sd | Adjusted $\beta$(95%CI) | Mean$\pm$sd | Adjusted $\beta$(95%CI) | Mean$\pm$sd | Adjusted $\beta$(95%CI) | Mean$\pm$sd | Adjusted $\beta$(95%CI) |
| 3 days | no (n=8） | 0.079±0.047 | - | 0.026±0.011 | - | 0.034±0.01 | - | 0.023±0.009 | - | 0.014±0.008 | - | 0.02±0.02 |  |
|  | yes (n=0） | - | - | - | - | - | - | - | - | - | - |  |  |
| 42 days | no (n=94） | 0.088±0.041 | ref | 0.039±0.018 | ref | 0.037±0.013 | ref | 0.025±0.012 | ref | 0.023±0.011 | ref | 0.02±0.01 | ref |
|  | yes (n=10) | 0.079±0.032 | -0.01(-0.04,0.02) | 0.034±0.012 | -0.01(-0.02,0.01) | 0.037±0.013 | -0.002(-0.01,0.01) | 0.021±0.008 | -0.01(-0.01,0.01) | 0.02±0.006 | -0.004(-0.01,0.003) | 0.02±0.01 | -0.001(-0.01,0.01) |
| 3 months | no (n=71） | 0.093±0.042 | ref | 0.04±0.019 | ref | 0.036±0.012 | ref | 0.025±0.011 | ref | 0.023±0.009 | ref | 0.02±0.01 | ref |
|  | yes (n=6) | 0.076±0.032 | -0.02(-0.06,0.02) | 0.033±0.006 | -0.01(-0.02,0.01) | 0.029±0.015 | -0.01(-0.02,0.004) | 0.023±0.009 | -0.001(-0.01,0.01) | 0.022±0.012 | 0.0002(-0.01,0.01) | 0.01±0.01 | -0.01(-0.02,0.004) |
| 6 months | no (n=70） | 0.105±0.042 | ref | 0.044±0.023 | ref | 0.037±0.012 | ref | 0.029±0.013 | ref | 0.023±0.012 | ref | 0.02±0.01 | ref |
|  | yes (n=6) | 0.093±0.031 | -0.01(-0.05,0.02) | 0.042±0.012 | -0.001(-0.02,0.02) | 0.034±0.011 | -0.003(-0.01,0.01) | 0.033±0.014 | 0.004(-0.01,0.01) | 0.032±0.013 | 0.01(-0.002,0.02) | 0.02±0.01 | -0.003(-0.02,0.01) |
| **Cesarean section** |  |  |  |  |  |  |  |  |  |  |  |  |  |
| 3 days | no (n=1） | 0.05 |  | 0.02 |  | 0.03 |  | 0.0002 |  | 0.02 |  | 0.0002 |  |
|  | yes (n=0） | - |  | - |  | - |  | - |  | - |  |  |  |
| 42 days | no (n=21） | 0.082±0.038 | ref | 0.04±0.017 | ref | 0.035±0.015 | ref | 0.021±0.011 | ref | 0.021±0.009 | ref | 0.02±0.02 | ref |
|  | yes (n=11) | 0.104±0.032 | 0.02(-0.01,0.04) | 0.043±0.018 | 0.003(-0.01,0.02) | 0.045±0.012 | 0.01(-0.003,0.02) | 0.024±0.012 | 0.001(-0.01,0.01) | 0.02±0.006 | -0.002(-0.01,0.005) | 0.03±0.01 | 0.01（-0.003，0.02） |
| 3 months | no (n=23） | 0.103±0.039 | ref | 0.043±0.02 | ref | 0.039±0.012 | ref | 0.028±0.013 | ref | 0.024±0.012 | ref | 0.03±0.02 | ref |
|  | yes (n=8) | 0.099±0.04 | -0.01(-0.04,0.03) | 0.039±0.013 | -0.01(-0.03,0.01) | 0.038±0.01 | -0.003(-0.01,0.01) | 0.027±0.01 | -0.001(-0.01,0.01) | 0.017±0.005 | -0.01(-0.02,0.001) | 0.02±0.01 | -0.004(-0.02,0.01) |
| 6 months | no (n=19） | 0.108±0.039 | ref | 0.045±0.021 | ref | 0.039±0.011 | ref | 0.032±0.012 | ref | 0.026±0.011 | ref | 0.02±0.02 | ref |
|  | yes (n=10) | 0.102±0.031 | -0.001(-0.03,0.03) | 0.041±0.015 | -0.003(-0.02,0.01) | 0.043±0.012 | 0.003(-0.01,0.01) | 0.026±0.01 | -0.004(-0.01,0.01) | 0.021±0.007 | -0.003(-0.01,0.01) | 0.03±0.01 | 0.003(-0.01,0.02) |

All regression models included infant age, sex (female, male) and feeding pattern (exclusive breast-feeding, mixed feeding) as covariates in the same models.


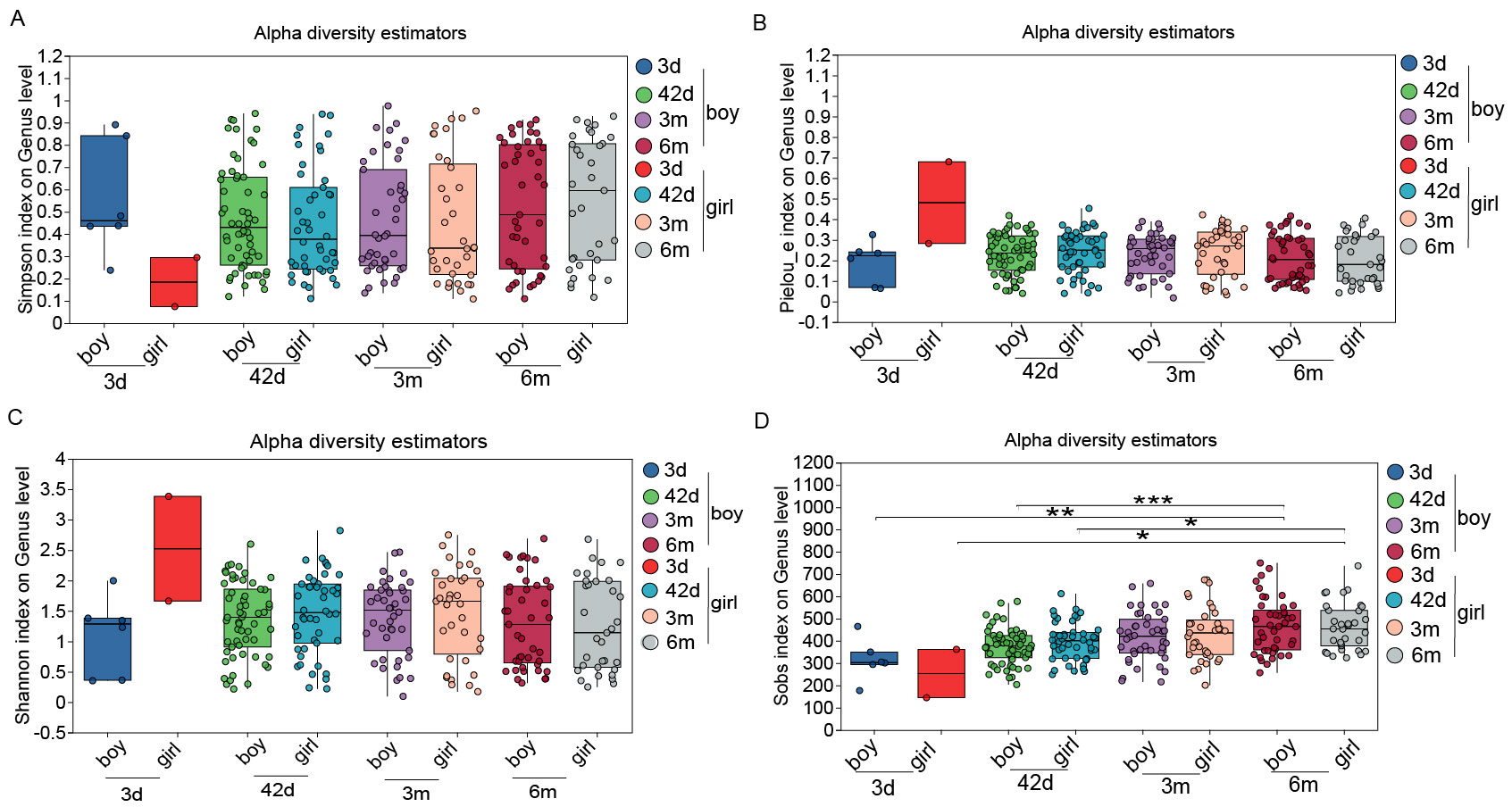


**Supplementary figure 1**. The Alpha diversity of gut microbiota from birth to age 6 months in boy and girl infants born by vaginal delivery. (A) Simpson index. (B) Pielou’s evenness index. (C) Shannon index. (D) Sobs index. Note: 3d, age 3 days; 42d, age 42 days; 3m, age 3 months; 6m, age 6 months. Only two girls aged 3 days. * *p*<0.05, ** *p*<0.01, *** *p*<0.001 for two groups comparison by Wilcoxon test.


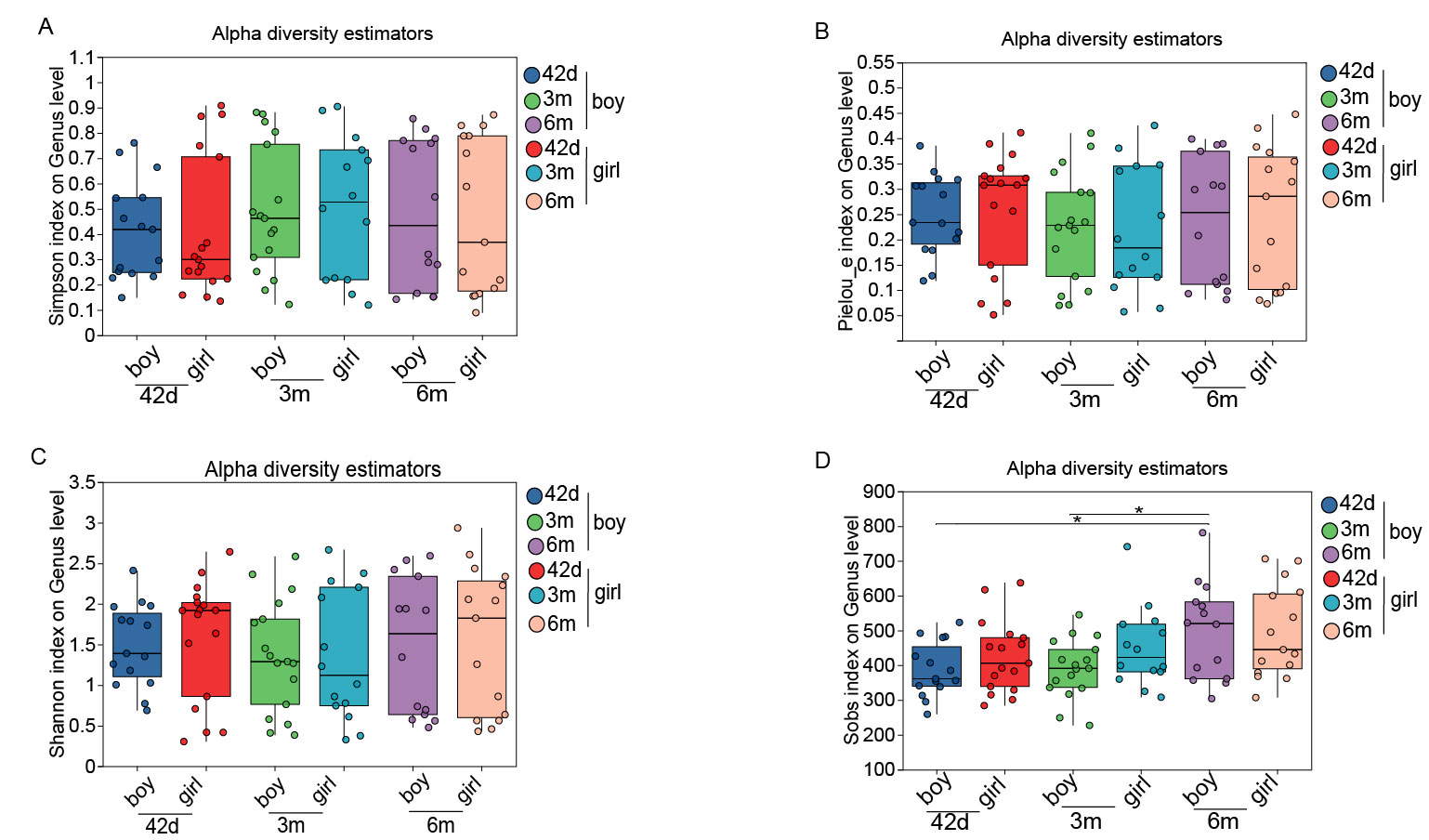


**Supplementary figure 2**. The Alpha diversity of gut microbiota from birth to age 6 months in boy and girl infants born by cesarean section. (A) Simpson index. (B) Pielou’s evenness index. (C) Shannon index. (D) Sobs index. Note: 3d, age 3 days; 42d, age 42 days; 3m, age 3 months; 6m, age 6 months. * *p*<0.05 for two groups comparison by Wilcoxon test.


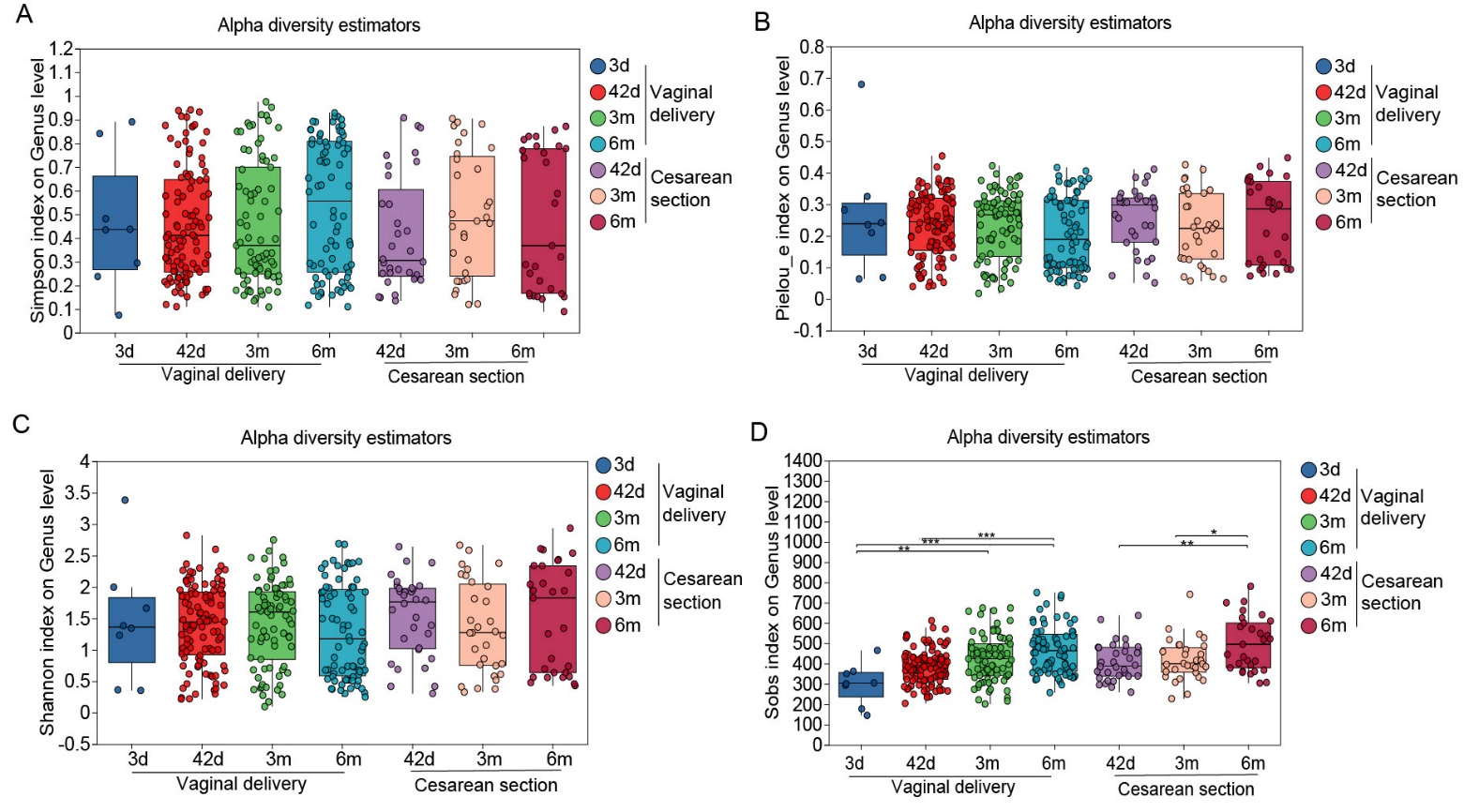


**Supplementary figure 3**. The Alpha diversity of gut microbiota from birth to age 6 months between infants born by vaginal delivery and those by cesarean section. (A) Simpson index. (B) Pielou’s evenness index. (C) Shannon index. (D) Sobs index. Note: 3d, age 3 days; 42d, age 42 days; 3m, age 3 months; 6m, age 6 months. * *p*<0.05, ** *p*<0.01, *** *p*<0.001 for two groups comparison by Wilcoxon test.


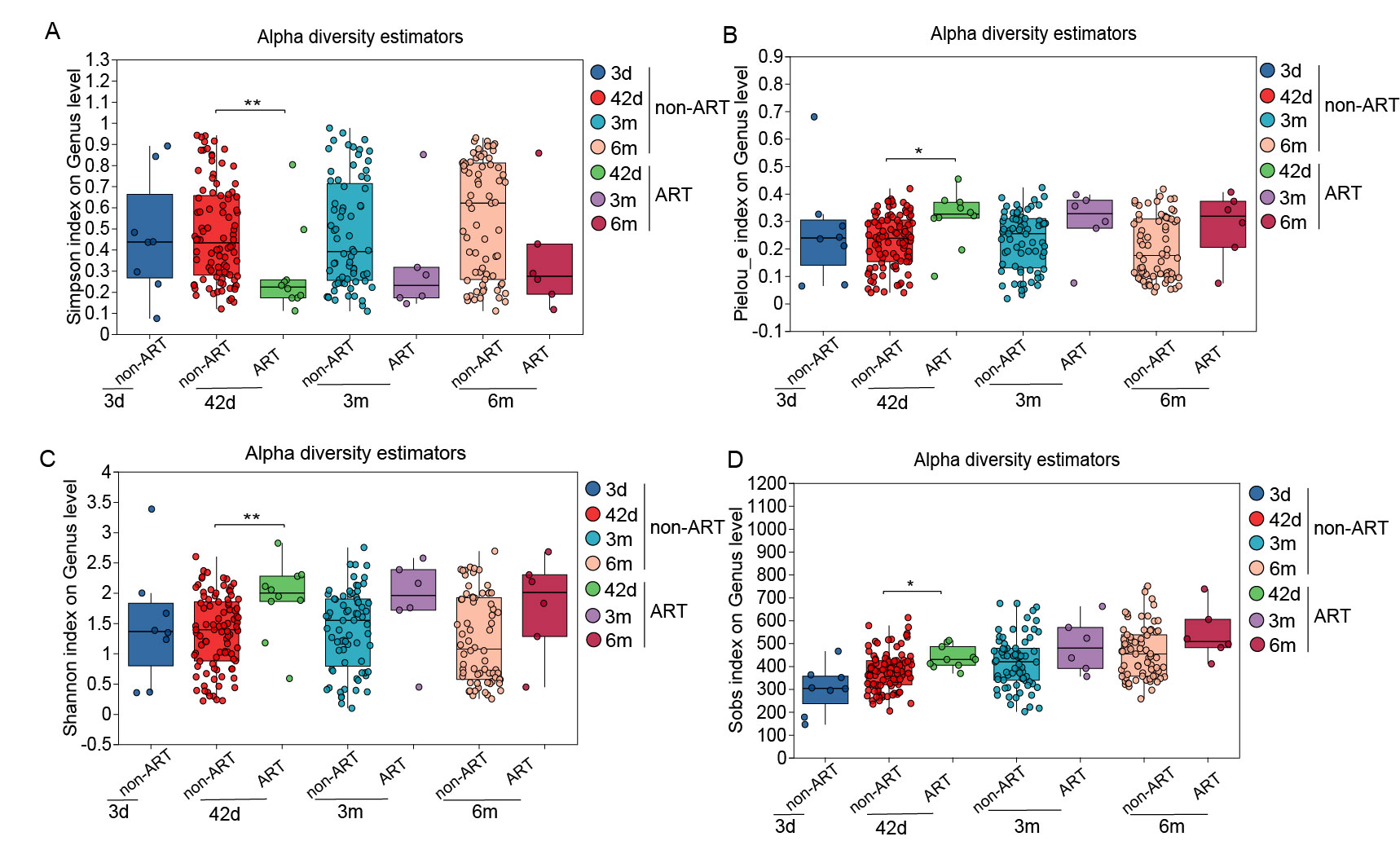


**Supplementary figure 4**. The Alpha diversity of gut microbiota from birth to age 6 months in infants with ART born by vaginal delivery. (A) Simpson index. (B) Pielou’s evenness index. (C) Shannon index. (D) Sobs index. Note: 3d, age 3 days; 42d, age 42 days; 3m, age 3 months; 6m, age 6 months. * *p*<0.05, ** *p*<0.01 for two groups comparison by Wilcoxon test.


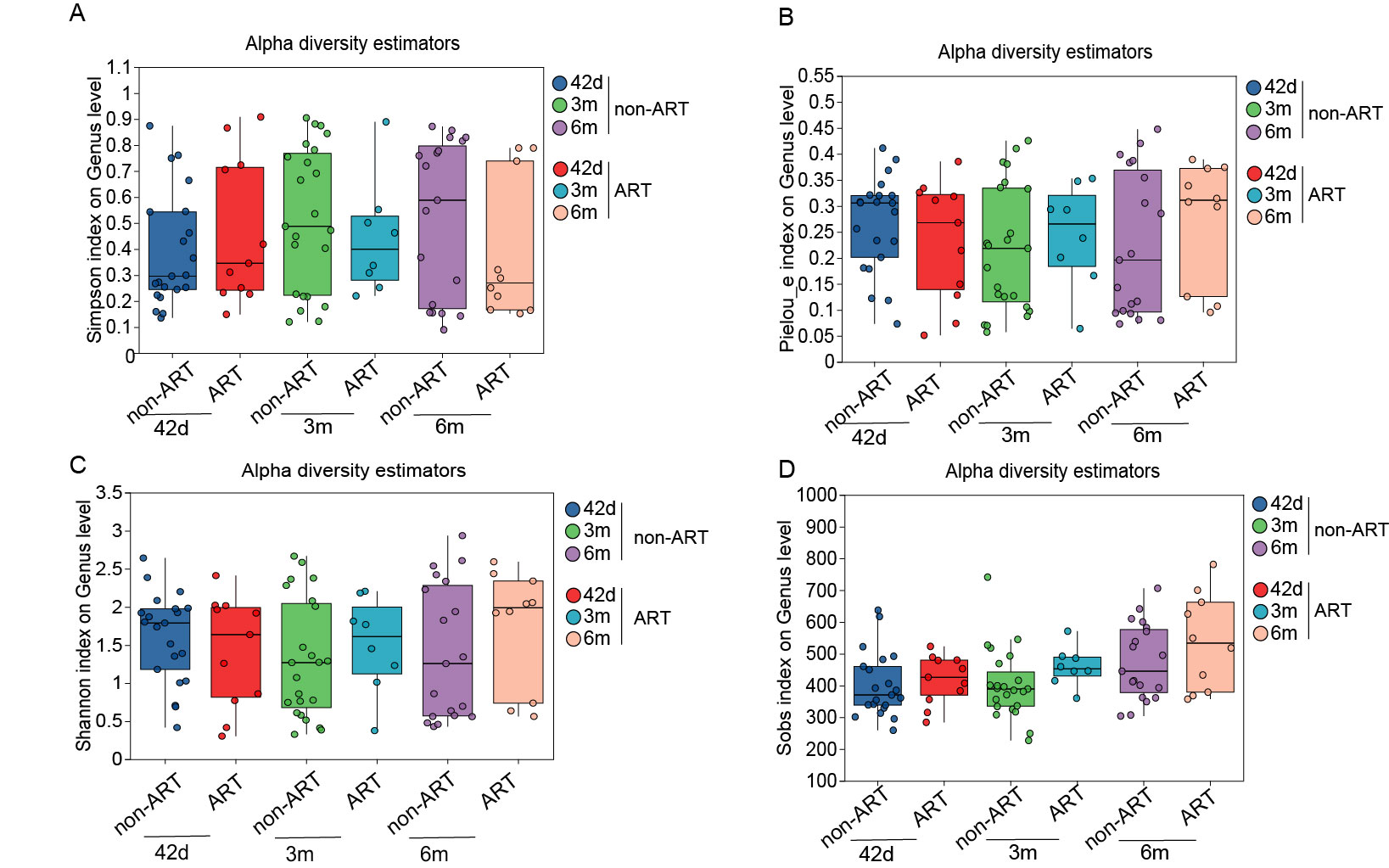


**Supplementary figure 5**. The Alpha diversity of gut microbiota from birth to age 6 months in infants with ART born by cesarean section. (A) Simpson index. (B) Pielou’s evenness index. (C) Shannon index. (D) Sobs index. Note: 3d, age 3 days; 42d, age 42 days; 3m, age 3 months; 6m, age 6 months.
